# Control of vein network topology by auxin transport

**DOI:** 10.1186/s12915-015-0208-3

**Published:** 2015-11-11

**Authors:** Carla Verna, Megan G. Sawchuk, Nguyen Manh Linh, Enrico Scarpella

**Affiliations:** Department of Biological Sciences, University of Alberta, Edmonton, Alberta Canada

**Keywords:** Arabidopsis, Leaf development, Vein network formation, Auxin, *PIN* genes

## Abstract

**Background:**

Tissue networks such as the vascular networks of plant and animal organs transport signals and nutrients in most multicellular organisms. The transport function of tissue networks depends on topological features such as the number of networks’ components and the components’ connectedness; yet what controls tissue network topology is largely unknown, partly because of the difficulties in quantifying the effects of genes on tissue network topology. We address this problem for the vein networks of plant leaves by introducing biologically motivated descriptors of vein network topology; we combine these descriptors with cellular imaging and molecular genetic analysis; and we apply this combination of approaches to leaves of *Arabidopsis thaliana* that lack function of, overexpress or misexpress combinations of four *PIN-FORMED* (*PIN*) genes—*PIN1*, *PIN5*, *PIN6*, and *PIN8*—which encode transporters of the plant signal auxin and are known to control vein network geometry.

**Results:**

We find that *PIN1* inhibits vein formation and connection, and that *PIN6* acts redundantly to *PIN1* in these processes; however, the functions of *PIN6* in vein formation are nonhomologous to those of *PIN1*, while the functions of *PIN6* in vein connection are homologous to those of *PIN1*. We further find that *PIN8* provides functions redundant and homologous to those of *PIN6* in *PIN1*-dependent inhibition of vein formation, but that *PIN8* has no functions in *PIN1*/*PIN6*-dependent inhibition of vein connection. Finally, we find that *PIN5* promotes vein formation; that all the vein-formation-promoting functions of *PIN5* are redundantly inhibited by *PIN6* and *PIN8*; and that these functions of *PIN5*, *PIN6*, and *PIN8* are independent of *PIN1*.

**Conclusions:**

Our results suggest that PIN-mediated auxin transport controls the formation of veins and their connection into networks.

**Electronic supplementary material:**

The online version of this article (doi:10.1186/s12915-015-0208-3) contains supplementary material, which is available to authorized users.

## Background

Signals and nutrients are transported within and between the organs of most multicellular organisms by tissue networks. What controls the formation of tissue networks is thus a central question in biology. In animals, many of these networks are stereotyped [1]; by contrast, the vein networks of plant leaves are both reproducible and variable [2, 3]. Consider, for example, the vein network of an *Arabidopsis thaliana* leaf [4–10]: lateral veins branch from a single midvein and connect to distal veins to form loops; minor veins branch from midvein and loops, and connect to other veins to form a mesh; and loops and minor veins curve near the leaf margin to lend a scalloped outline to the vein network. Geometric features of the vein network such as these are reproducible from leaf to leaf—so much so that they are used as a taxonomic characteristic (e.g., [11]). By contrast, topological features of the vein network are variable [6, 7, 10, 12–14]: the number of veins differs from leaf to leaf, and whether a vein will connect to another vein on both ends or one end will terminate free of contact with other veins is unpredictable; this is always so for minor veins, but even loops can occasionally fail to connect to other veins at one end.

While no evidence is available that associates geometric features of vein networks with the networks’ functional traits, abundant evidence exists that associates functional traits of vein networks with the networks’ topological features (reviewed in [15, 16]); yet our knowledge of the signals that control vein network topology is limited, and most such signals also control vein network geometry (e.g., [17–23]). One of very few exceptions [24–26] is the control of vein network topology by intracellular transport of the plant signal auxin suggested by genetic evidence: leaves of double mutants in the genes encoding the endoplasmic-reticulum (ER)-localized PIN-FORMED6 (PIN6) and PIN8 auxin transporters of Arabidopsis [27–33] have higher vein-density [33]; the vein density defect of *pin6*;*pin8* leaves is suppressed by mutation of the gene encoding the ER-localized PIN5 auxin transporter [28, 33, 34]; overexpression of *PIN6* or *PIN8* results in lower vein-density, and overexpression of *PIN5* results in the opposite defect [33].

In contrast to the control of vein network topology by intracellular auxin transport, no genetic evidence is available in support of a role for the cell-to-cell transport of auxin in control of vein network topology; yet such a role seems to be suggested by imaging and inhibitor studies. Expression of the PIN1 auxin efflux protein [27, 35] is initiated in broad domains of leaf inner cells that become gradually restricted to files of vascular precursor cells in contact with pre-existing, narrow PIN1 expression domains [33, 36–42]. Within broad expression domains, PIN1 is localized isotropically—or nearly so—to the plasma membrane (PM) of leaf inner cells. As expression of PIN1 becomes gradually restricted to files of vascular precursor cells, PIN1 localization becomes polarized to the side of the PM facing the pre-existing, narrow PIN1 expression domains with which the narrowing domains are in contact. Initially, PIN1 expression domains are in contact with pre-existing domains at one end only, but they can eventually become connected to other PIN1 expression domains at both ends. Inhibitors of cellular auxin efflux delay the restriction of PIN1 expression domains and the polarization of PIN1 localization [38, 39], and induce the formation of more veins [4, 5].

The polar localization of PIN1 to the PM of vascular cells—toward pre-existing veins and ultimately the root tip—is thought to determine the polarity of intercellular auxin transport [43]: from the immature shoot-organs, where auxin is produced in large amounts [44, 45], to the roots [46, 47]. By contrast, the directions of ER-PIN-mediated intracellular auxin-transport are unclear. Available evidence suggests that PIN5 transports auxin from the cytoplasm to the ER lumen [28, 33], and that PIN6 and PIN8 transport it from the ER lumen to the cytoplasm or the nucleus [29–33], the envelope of which is continuous with the ER membrane [48, 49]; alternatively, PIN5, PIN6, and PIN8 could transport in the same direction but have different affinities for different auxins with different developmental functions (e.g., [50]).

Here we asked whether PIN1-mediated intercellular auxin-transport controlled vein network topology and, if so, whether it interacted with the control of vein network topology by ER-PIN-mediated intracellular auxin-transport. To address this question, we introduced descriptors of vein network topology that enable quantification of vein number, connectedness and continuity, and combined these topological descriptors with cellular imaging and molecular genetic analysis to quantify the contribution of *PIN1*, *PIN5*, *PIN6*, and *PIN8* to vein network topology. We derived cellular expression and genetic interaction maps of these genes in vein network formation, and suggest that the interaction between PIN1-mediated intercellular auxin-transport and ER-PIN-mediated intracellular auxin-transport controls the formation of veins and their connection into networks.

## Results and discussion

### Expression of *PIN1*, *PIN5*, *PIN6*, and *PIN8* during leaf development

**Fig. 1:**
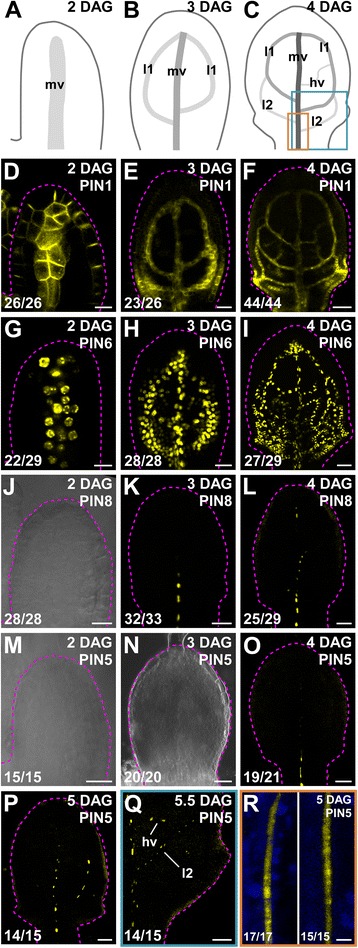
Expression of *PIN1*, *PIN5*, *PIN6*, and *PIN8* of Arabidopsis during first leaf development. **a**-**r**. *Top right*: leaf age in days after germination (DAG) and expression-reported gene (**d-r**). **d**-**r**. *Bottom left*: reproducibility index. **a**-**c**. Midvein, loops and minor veins form sequentially during leaf development [4, 5, 12, 13]; increasingly darker gray depicts progression through successive stages of vein development. *Boxes* in (**c**) illustrate positions of close-ups in (**q**) (cyan) and (**r**) (orange). **d**-**r**. Confocal laser scanning microscopy with (**j**,**m**,**n**) or without (**d**-**i**,**k**,**l**,**o**-**r**) transmitted light; first leaves. Yellow: expression of PIN1::PIN1:GFP (**d**-**f**), PIN6::YFPnuc (**g**-**i**), PIN8::YFPnuc (**j**-**l**), PIN5::YFPnuc (**m**-**q**), PIN5::PIN5:GFP^MGS^ (**r**, *left*) or PIN5::PIN5:GFP^AG^ (**r**, *right*). Blue: autofluorescence (**r**). Dashed magenta line delineates leaf primordium outline. hv, minor veins; l1, first loop; l2, second loop; mv, midvein. Bars: (**d**,**g**,**j**,**m**,**r**) 10 μm; (**e**,**h**,**k**,**n**) 25 μm; (**f**,**i**,**l**,**o**-**q**) 50 μm

Veins form sequentially during Arabidopsis leaf development: the formation of the midvein is followed by the formation of the first loops of veins (“first loops”), which in turn is followed by the formation of second loops and minor veins [4, 5, 12, 13] (Fig. 1a-c).

Two distinct auxin-transport pathways have overlapping functions in control of Arabidopsis vein-network geometry [33]. One pathway—mediated by the PM-localized PIN1 protein—transports auxin intercellularly [27, 35]; the other pathway—mediated by the ER-localized PIN5, PIN6, and PIN8 proteins—transports auxin intracellularly [27–34].

Consistent with their role in control of vein network geometry [4, 33, 51, 52], *PIN1* (AT1G73590), *PIN6* (AT1G77110), and *PIN8* (AT5G15100) are expressed in developing veins, although with different dynamics: expression of *PIN1* and *PIN6* is initiated in broad domains of leaf inner cells, domains that over time become restricted to single files of vascular precursor cells [33, 38–40] (Fig. 1d-i); by contrast, *PIN8* expression is restricted from early on to single files of leaf vascular cells [33] (Fig. 1j-l). It remains unclear, however, whether these different dynamics of *PIN* expression comprise onset of *PIN* expression at different stages of leaf development.

To address this question, we compared expression of *PIN1*, *PIN6*, and *PIN8* in first leaves 2, 3 and 4 days after germination (DAG). To visualize *PIN* expression, we used functional translational fusions (*PIN* promoter driving expression of the respective PIN:reporter fusion protein) [33, 37, 53] or transcriptional fusions (*PIN* promoter driving expression of a reporter protein) [33] (Additional file 1: Table S1); whenever we used transcriptional fusions, their expression matched that of the respective, functional translational fusions [33] (Additional file 2: Figure S1), suggesting that those *PIN* promoters contain all the regulatory elements required for functional expression of the respective genes.

While expression of a PIN1::PIN1:GFP translational fusion (*PIN1* promoter driving expression of PIN1:GFP fusion protein) and of a PIN6::YFPnuc transcriptional fusion (*PIN6* promoter driving expression of a nuclear yellow fluorescent protein) was already visible 2 DAG (Fig. 1d, g), expression of PIN8::YFPnuc was first detected 3 DAG (Fig. 1j, k), suggesting that *PIN8* expression is initiated after the onset of expression of both *PIN1* and *PIN6*.

*PIN5* (AT5G16530) is expressed in veins of mature leaves [28, 54], but its expression during leaf development is unknown. Transcriptional and translational fusions of *PIN5* are expressed in similar domains [28, 34, 54], suggesting that the *PIN5* promoter contains all the regulatory elements required for *PIN5* expression. Thus, to visualize *PIN5* expression during leaf development, we imaged PIN5::YFPnuc expression in first leaves 2, 3, 4, 5 and 5.5 DAG.

Expression of PIN5::YFPnuc was first detected in the midvein of 4-DAG leaves (Fig. 1m-o); at 5 DAG, PIN5::YFPnuc was additionally expressed in first loops (Fig. 1p), and at 5.5 DAG PIN5::YFPnuc was additionally expressed in second loops and minor veins (Fig. 1q). Thus our results suggest that *PIN5* expression is initiated after *PIN8* expression (Fig. 1k, n, o) and that, as *PIN8*, *PIN5* is expressed from early on in single files of leaf vascular cells (Fig. 1k, l, o-q).

Expression of *PIN5* in single files of leaf vascular cells—suggested by PIN5::YFPnuc expression—was supported by expression of two functional (Additional file 1: Table S1) [34] PIN5::PIN5:GFP translational fusions (Fig. 1r).

### Expression of *PIN1*, *PIN5*, *PIN6*, and *PIN8* in leaf vascular cells

Because *PIN1*, *PIN5*, *PIN6*, and *PIN8* are all expressed in developing veins (Fig. 1), we asked whether these genes were expressed in the same vascular cells. To address this question, we imaged pairwise combinations of fluorescent reporters of *PIN1*, *PIN5*, *PIN6*, and *PIN8* in midvein cells of 4-DAG first leaves—where these genes are expressed (Fig. 1f, i, l, o)—and quantified reporter coexpression.

**Fig. 2:**
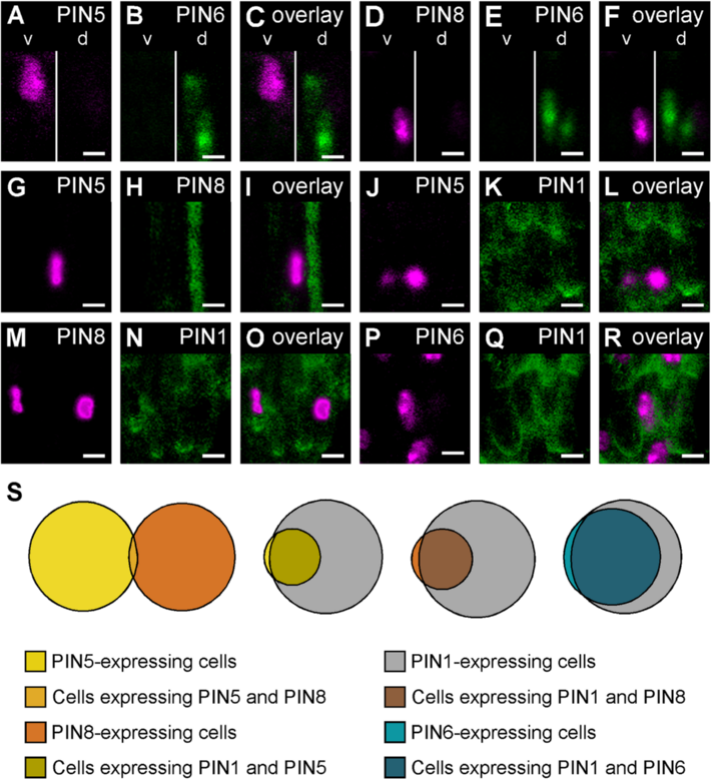
Expression of *PIN1*, *PIN5*, *PIN6*, and *PIN8* in leaf vascular cells. **a**-**r**. *Top right*: expression-reported gene. Confocal laser scanning microscopy; first leaves. **a**-**r**. Expression of PIN5::YFPnuc (**a**,**g**,**j**), PIN6::CFPnuc (**b**,**e**), PIN8::YFPnuc (**d**,**m**), PIN8::PIN8:GFP^MGS^ (**h**), PIN1::PIN1:GFP (**k**,**n**,**q**), PIN6::YFPnuc (**p**), and respective overlays (**c**,**f**,**i**,**l**,**o**,**r**). **s**. Proportional Venn diagrams of percentage of cells expressing fluorescent reporters in 25-μm by 25-μm midvein regions of 4-day-old first leaves (one region per midvein) in different pairwise combinations of reporters. Sample population sizes: PIN5::YFPnuc;PIN8::PIN8:GFP^MGS^, 40 leaves (41 PIN5::YFPnuc-expressing cells; 39 PIN8::PIN8:GFP^MGS^-expressing cells); PIN5::YFPnuc;PIN1::PIN1:GFP, 26 leaves (30 PIN5::YFPnuc-expressing cells; 119 PIN1::PIN1:GFP-expressing cells); PIN8::YFPnuc;PIN1::PIN1:GFP, 25 leaves (34 PIN8::YFPnuc-expressing cells; 122 PIN1::PIN1:GFP-expressing cells); PIN6::YFPnuc;PIN1::PIN1:GFP, 31 leaves (127 PIN6::YFPnuc-expressing cells; 174 PIN1::PIN1:GFP-expressing cells). d, dorsal focal plane; v, ventral focal plane. Bars: (**a-r**) 5 μm

In none of the 20 analyzed leaves coexpressing PIN5::YFPnuc and PIN6::CFPnuc (*PIN6* promoter driving expression of a nuclear cyan fluorescent protein) were cells expressing PIN5::YFPnuc ever on the same plane as cells expressing PIN6::CFPnuc: cells expressing PIN5::YFPnuc were located ventrally, while cells expressing PIN6::CFPnuc were located dorsally (Fig. 2a-c). Likewise, in none of the 20 analyzed leaves coexpressing PIN8::YFPnuc and PIN6::CFPnuc were cells expressing PIN8::YFPnuc ever on the same plane as cells expressing PIN6::CFPnuc: cells expressing PIN8::YFPnuc were located ventrally, while cells expressing PIN6::CFPnuc were located dorsally (Fig. 2d-f). And although cells expressing PIN5::YFPnuc or PIN8::PIN8:GFP^MGS^ were both on the same ventral plane (Fig. 2g-i), only fewer than 3 % of the cells expressing either reporter expressed both (Fig. 2s).

Approximately 95 % of PIN5::YFPnuc-expressing cells expressed PIN1::PIN1:GFP, but only ~25 % of the PIN1::PIN1:GFP-expressing cells that were on the same ventral plane as cells expressing PIN5::YFPnuc expressed this reporter (Fig. 2j-l, s). Likewise, ~90 % of PIN8::YFPnuc-expressing cells expressed PIN1::PIN1:GFP, but only ~25 % of the PIN1::PIN1:GFP-expressing cells that were on the same ventral plane as cells expressing PIN8::YFPnuc expressed this reporter (Fig. 2m-o, s). Finally, consistent with previous observations [33], ~95 % of PIN6::YFPnuc-expressing cells expressed PIN1::PIN1:GFP, and ~75 % of the PIN1::PIN1:GFP-expressing cells that were on the same dorsal plane as cells expressing PIN6::YFPnuc expressed this reporter (Fig. 2p-s).

Thus our results suggest that *PIN5*, *PIN6*, and *PIN8* are expressed in mutually exclusive domains of leaf vascular cells, and that the *PIN1* cellular-expression domain overlaps with—but extends beyond—the *ER-PIN* cellular-expression domain.

### Unique and redundant functions of *PIN1*, *PIN5*, *PIN6*, and *PIN8* in control of vein network topology

*PIN1*, *PIN5*, *PIN6,* and *PIN8* control vein network geometry [4, 33, 51, 52]; we asked what their functions are in control of vein network topology.

To characterize vein network topology, we derived (*see*Methods and Additional file 3: Figure S2 for details) and used three descriptors based on numerical graph invariants: a cardinality index, a continuity index, and a connectivity index.

**Fig. 3:**
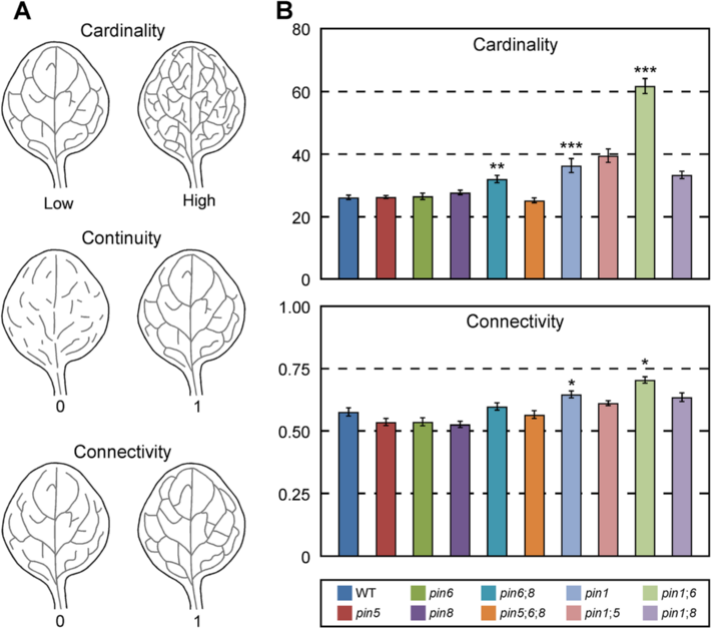
Functions of *PIN1*, *PIN5*, *PIN6*, and *PIN8* in control of vein network topology. **a**. Schematics of vein networks with low or high cardinality index (*top row*), minimum—i.e. 0—or maximum—i.e. 1—continuity index (*middle row*), or minimum—i.e. 0—or maximum—i.e. 1—connectivity index (*bottom row*). **b**. First leaves. Indices are expressed as mean ± SE. Difference between *pin6*;*8* and WT cardinality indices, between *pin1* and WT cardinality indices, between *pin1*;*6* and *pin1* cardinality indices, between *pin1* and WT connectivity indices, and between *pin1*;*6* and *pin1* connectivity indices was significant at *P* < 0.05 (*), *P* < 0.01 (**), or *P* < 0.001 (***) by *F*-test and *t*-test with Bonferroni correction. Sample population sizes: WT, 30; *pin5*, 30; *pin6*, 30; *pin8*, 27; *pin6*;*8*, 28; *pin5*;*6*;*8*, 28; *pin1*, 45; *pin1*;*5*, 57; *pin1*;*6*, 47; *pin1*;*8*, 37

The cardinality index is a proxy for the number of “veins” (i.e. stretches of vascular elements that contact other stretches of vascular elements at least at one of their two ends) in a network (Fig. 3a).

The continuity index quantifies how close a vein network is to a network with the same number of veins but in which at least one end of each “vein fragment” (i.e. a stretch of vascular elements that are free of contact with other stretches of vascular elements) contacts a vein. The continuity index ranges from 0—for a network of sole vein fragments—to 1—for a network without vein fragments (Fig. 3a).

The connectivity index quantifies how close a vein network is to a network with the same number of veins but in which both ends of each vein or vein fragment contact other veins. The connectivity index ranges from 0—for a network of “open” veins (i.e. veins that contact vein fragments or other veins only at one end)—to 1—for a network of “closed” veins (i.e. veins that contact vein fragments or other veins at both ends) (Fig. 3a).

Although the number of veins in a leaf is variable and it is unpredictable whether a developing vein will remain open at maturity [6, 7, 10, 12–14], the cardinality and connectivity indices of vein networks in different populations of WT leaves grown in identical conditions were reproducible (Figs. 3, 6, 7 and 8). This observation suggests that while the outcome of vein formation events is unpredictable for single veins, it is predictable—within the limits of statistical variation—for networks of veins. Thus—as for non-stereotyped animal-networks (reviewed in [55])—topology descriptors such as the cardinality and connectivity indices can be compared statistically across genotypes and conditions to identify reproducible patterns and their controls.

The continuity index of vein networks in different populations of WT leaves grown in identical conditions was also reproducible (Additional file 4: Figure S3)—a finding consistent with the stringent requirement for continuity of tissue systems with transport function, such as vein networks, and with the successful use of vein fragmentation as diagnostic criterion for the identification of mutants in genetic screens [18, 56, 57].

The continuity index of none of the mutants or transgenics in our study was different from that of WT (Additional file 4: Figure S3), suggesting that *PIN1*, *PIN5*, *PIN6*, and *PIN8* have no function in control of vein continuity or their functions in this process are redundant.

Consistent with previous observations [33], the vein network topology of *pin5*, *pin6*, or *pin8* was no different from that of WT (Fig. 3b); by contrast, the cardinality and connectivity indices of *pin1* vein networks were higher than those of WT vein networks (Fig. 3b), suggesting that *PIN1* inhibits the formation of veins and their connection.

We next asked whether *PIN5*, *PIN6*, or *PIN8* acted redundantly with *PIN1* in inhibition of vein formation and connection. The vein network topology of neither *pin1*;*pin5* (*pin1*;*5* hereafter) nor *pin1*;*8* differed from that of *pin1* (Fig. 3b); however, the cardinality and connectivity indices of *pin1*;*6* vein networks were higher than those of *pin1* vein networks (Fig. 3b), suggesting that *PIN6* acts redundantly with *PIN1* in inhibition of vein formation and connection.

**Fig. 4:**
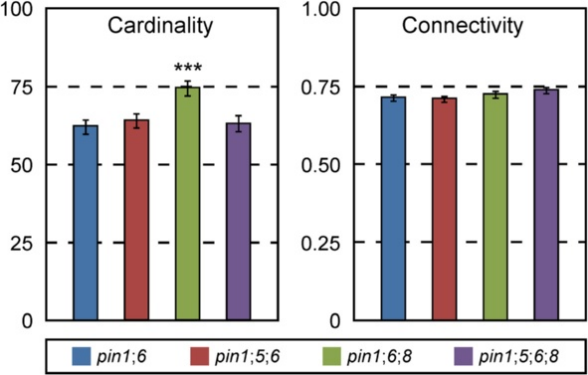
Functions of *PIN5* and *PIN8* in *PIN1*/*PIN6*-dependent control of vein network topology. First leaves. Indices are expressed as mean ± SE. Difference between *pin1*;*6*;*8* and *pin1*;*6* cardinality indices was significant at *P* < 0.001 (***) by *F*-test and *t*-test with Bonferroni correction. Sample population sizes: *pin1*;*6*, 103; *pin1*;*5*;*6*, 104; *pin1*;*6*;*8*, 98; *pin1*;*5*;*6*;*8*, 109

Next, we asked whether *PIN5* or *PIN8* acted redundantly with *PIN6* in *PIN1*-dependent inhibition of vein formation and connection. The vein network topology of *pin1*;*5*;*6* was no different from that of *pin1*;*6* (Fig. 4), but the cardinality index of *pin1*;*6*;*8* vein networks was higher than that of *pin1*;*6* vein networks (Fig. 4), suggesting that *PIN8* acts redundantly with *PIN6* in *PIN1*-dependent inhibition of vein formation; by contrast, the connectivity index of *pin1*;*6*;*8* vein networks was no different from that of *pin1*;*6* vein networks (Fig. 4), suggesting that *PIN8* has no function redundant to that of *PIN6* in *PIN1*-dependent inhibition of vein connection. Because the vein network topology of neither *pin6* nor *pin8* differs from that of WT (Fig. 3b), but the cardinality index of *pin6*;*8* vein networks is higher than that of WT (Fig. 3b), *PIN6* and *PIN8* also have redundant functions in inhibition of vein formation that are independent of *PIN1*. Thus the enhancement of *pin1*;*6* cardinality defects by *PIN8* could be interpreted as the result of the simultaneous loss of the *PIN1*-dependent pathway and of the parallel, *PIN6*/*PIN8*-dependent, *PIN1*-independent pathway—rather than evidence that *PIN8* acts redundantly with *PIN6* in *PIN1*-dependent inhibition of vein formation. However, we do not favor this interpretation because the cardinality defect of *pin1*;*6*;*8* is much greater than the sum of the cardinality defects of *pin1* and *pin6*;*8*.

We finally asked whether *PIN5* acted redundantly with *PIN6* and *PIN8* in *PIN1*-dependent or *PIN1*-independent inhibition of vein formation. The vein network topology of *pin1*;*5*;*6*;*8* was no different from that of *pin1*;*6* (Fig. 4) and that of *pin5*;*6*;*8* was no different from that of WT (Fig. 3b), suggesting that *pin5* suppresses the effects of *pin6* and *pin8* on *PIN1*-independent inhibition of vein formation. In agreement with interpretations of similar genetic interactions in other organisms (e.g., [58–60]), the most parsimonious account for our observations is that *PIN5* promotes vein formation; that *PIN6* and *PIN8* redundantly and completely inhibit *PIN5*-dependent promotion of vein formation; and that these functions of *PIN5*, *PIN6*, and *PIN8* are independent of *PIN1*. Further, because expression of *PIN5* and *PIN8* is initiated at post-formative stages of vein development [33] (Fig. 1), these genes most likely control vein formation indirectly—for example, through feedback on vascular precursor cells located in more-immature parts of the leaf (e.g., [25]; reviewed in [61, 62]). Finally, because *PIN5*, *PIN6*, and *PIN8* are expressed in non-overlapping sets of vascular cells (Fig. 2), the genetic interaction between these genes—as that between other genes expressed in mutually exclusive domains (e.g., [63–67] and references therein)—presumably reflects underlying cell-cell interactions.

### Redundant functions of *PIN1*, *PIN6*, and *PIN8* in control of auxin distribution in developing leaves

*PIN1* inhibits vein formation, and *PIN6* acts redundantly with *PIN1* in inhibition of vein formation and with *PIN8* in *PIN1*-independent inhibition of vein formation (Figs. 3 and 4). We asked whether such redundancy extended to control of auxin distribution in developing leaves, which is known to control vein formation [4, 5, 23, 33, 38, 39, 68]. To address this question, we imaged expression of the auxin reporter DR5rev::YFPnuc [33, 36, 69] in 4-DAG first leaves of WT, *pin6*;*8*, *pin1*, and *pin1*;*6*.

**Fig. 5:**
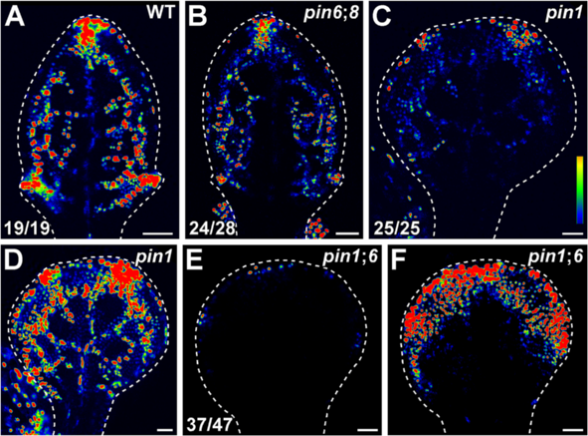
Expression of DR5rev::YFPnuc in *pin* developing leaves. **a**-**d**. Confocal laser scanning microscopy; first leaves 4 days after germination. Look-up table (ramp in **c**) visualizes expression levels. *Top right*: genotype. *Bottom left*: reproducibility index. *Dashed white line* delineates leaf primordium outline. Images in **a**, **b**, **c** and **e** were taken at identical settings and show increasingly weaker DR5rev::YFPnuc expression in *pin6*;*8*, *pin1*, and *pin1*;*6*. Images in **a**, **d** and **f** were taken by matching signal intensity to detector’s input range (~4 % saturated pixels), and show increasingly broader DR5rev::YFPnuc expression domains in *pin1* and *pin1*;*6*. Bars: (**a-f**) 50 μm

As previously reported [22, 33, 38, 68], in WT the DR5 promoter was strongly active in narrow domains that coincide with sites of vein formation (Fig. 5a). Consistent with previous observations [29–33], DR5rev::YFPnuc expression was weaker in *pin6*;*8* than in WT, but domains of DR5rev::YFPnuc expression were equally narrow in *pin6*;*8* and WT (Fig. 5a, b). Levels of DR5rev::YFPnuc expression were lower, and domains of DR5rev::YFPnuc expression were broader, in *pin1* than in WT or *pin6*;*8* (Fig. 5a-d); and DR5rev::YFPnuc expression levels were even lower, and DR5rev::YFPnuc expression domains even broader, in *pin1*;*6* (Fig. 5c-f).

Thus our results suggest that the redundancy between *PIN1*, *PIN6*, and *PIN8* that underlies control of vein formation extends to control of auxin distribution in developing leaves (see Conclusions).

### Homologous and nonhomologous functions of *PIN1* and *PIN6* in vein network formation

*PIN6* acts redundantly with *PIN1* in control of vein network geometry [33] and topology (Fig. 3b); however, the redundancy between *PIN1* and *PIN6* is unequal: the geometry and topology of *pin6* vein networks are no different from those of WT vein networks but those of *pin1* vein networks are, suggesting that *PIN1* can provide all—or nearly all—the functions of *PIN6* in vein network formation and that, by contrast, *PIN6* is unable to provide all the functions of *PIN1* in this process. Such unequal redundancy could reflect nonhomologous functions of *PIN1* and *PIN6* in vein network formation—a possibility consistent with the different localization of PIN1 and PIN6: PIN1 is predominantly localized to the PM [35], while PIN6 is predominantly localized to the ER [33]. On the other hand—at least in other organisms—redundant, homologous functions can be provided by proteins that are localized to different cellular compartments (e.g., [70, 71] and references therein). Further, at least some of the functions of *PIN1* in vein network formation depend on *PIN1* expression in leaf epidermal cells [51, 72]—leaf epidermal cells that fail, by contrast, to express *PIN6* [33] (Fig. 1). Thus the unequal redundancy of *PIN1* and *PIN6* in vein network formation could alternatively be accounted for by their different expression domains.

To test these possibilities, we used the promoter of the *RIBOSOMAL PROTEIN S5A* (*RPS5A*) gene (AT3G11940)—highly active in developing organs, including their epidermal cells [73]—to express *PIN1* (RPS5A::PIN1) or *PIN6* (RPS5A::PIN6) in the *pin1* background, and compared phenotype features of RPS5A::PIN1;*pin1* and RPS5A::PIN6;*pin1* with those of *pin1* and WT.

**Fig. 6:**
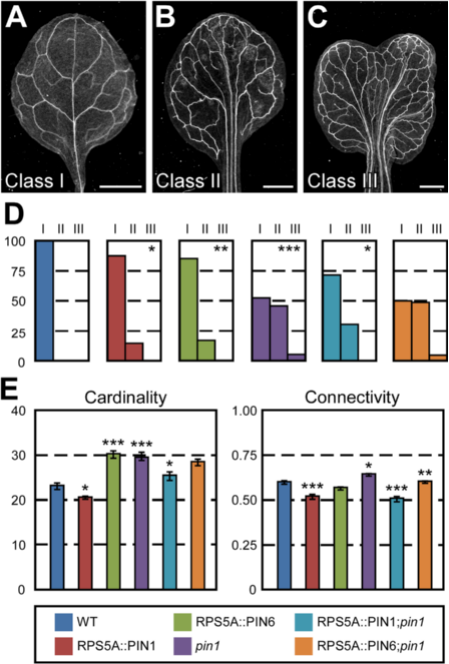
Functions of *PIN1* and *PIN6* in vein network formation. **a**-**c**. Dark-field illumination of mature first leaves illustrating phenotype classes: unbranched, narrow midvein and scalloped vein-network outline (**a**); bifurcated midvein and scalloped vein-network outline (**b**); fused leaves with scalloped vein-network outline (**c**). **d**. Percentages of leaves in phenotype classes. Difference between RPS5A::PIN1 and WT, between RPS5A::PIN6 and WT, between *pin1* and WT, and between RPS5A::PIN1;*pin1* and *pin1* was significant at *P* < 0.05 (*), *P* < 0.01 (**), or *P* < 0.001 (***) by Kruskal-Wallis and Mann–Whitney test with Bonferroni correction. Sample population sizes: WT, 65; RPS5A::PIN1, 55; RPS5A::PIN6, 58;*pin1*, 116; RPS5A::PIN1;*pin1*, 71; RPS5A::PIN6;*pin1*, 140. **e**. First leaves. Indices are expressed as mean ± SE. Difference between RPS5A::PIN1 and WT cardinality indices, between RPS5A::PIN6 and WT cardinality indices, between *pin1* and WT cardinality indices, between RPS5A::PIN1;*pin1* and *pin1* cardinality indices, between RPS5A::PIN1 and WT connectivity indices, between *pin1* and WT connectivity indices, between RPS5A::PIN1;*pin1* and *pin1* connectivity indices, and between RPS5A::PIN6;*pin1* and *pin1* connectivity indices was significant at *P* < 0.05 (*), *P* < 0.01 (**), or *P* < 0.001 (***) by *F*-test and *t*-test with Bonferroni correction. Sample population sizes as in (**d**). Bars: (**a**-**c**) 1 mm

We first asked whether *PIN6* could provide functions in control of vein network geometry homologous to those of *PIN1*. The geometry of ~15 % of the vein networks of RPS5A::PIN1 and RPS5A::PIN6, and—as previously reported [33]—of nearly 50 % of *pin1* vein networks was abnormal (Fig. 6a-d). RPS5A::PIN1 shifted the spectrum of vein network geometries of *pin1* toward the vein network geometry of WT but RPS5A::PIN6 failed to do so (Fig. 6a-d), suggesting that *PIN6* is unable to provide functions in control of vein network geometry homologous to those of *PIN1*.

We next asked whether *PIN6* could provide functions in control of vein network topology homologous to those of *PIN1*. The cardinality and connectivity indices of RPS5A::PIN1 vein networks were lower than those of WT vein networks (Fig. 6e), supporting that *PIN1* inhibits vein formation and connection. The cardinality index of RPS5A::PIN6 vein networks was higher than that of WT vein networks (Fig. 6e), suggesting that ectopic expression of *PIN6* in the epidermis promotes vein formation. As reported above (Fig. 3b), the cardinality and connectivity indices of *pin1* vein networks were higher than those of WT vein networks (Fig. 6e). RPS5A::PIN1 shifted the cardinality index of *pin1* vein networks toward that of WT vein networks but RPS5A::PIN6 failed to do so (Fig. 6e), suggesting that *PIN6* is unable to provide functions in vein formation homologous to those of *PIN1*. By contrast, both RPS5A::PIN1 and RPS5A::PIN6 shifted the connectivity index of *pin1* vein networks toward that of WT vein networks (Fig. 6e), suggesting that *PIN6* can provide functions in vein connection homologous to those of *PIN1*. Interpretations of similar genetic interactions in other organisms (e.g., [74–76]) suggest that the suppression of vein connectedness defects of *pin1* by RPS5A::PIN6 can be accounted for by at least two mechanisms. One possibility is that *PIN6* acts downstream of *PIN1* in the same pathway that controls vein connection; we do not favor this hypothesis, however, because it fails to predict the observed (Figs. 3 and 4) enhancement of vein connectedness defects of *pin1* by *pin6*. Alternatively, vein connection may be unfavored at high auxin levels [77], which would be the result of at least two separate pathways: PIN1-mediated auxin transport toward sites of vein formation [38–41] (Fig. 5) and PIN6-mediated increase in auxin levels within developing vascular cells [31–33] (Fig. 5) (see Conclusions).

In addition to vein network formation, *PIN6* acts redundantly with *PIN1* in cotyledon patterning, and as in vein network formation, the redundancy between *PIN1* and *PIN6* in cotyledon patterning is unequal [33]. We thus asked whether *PIN6* could provide functions in cotyledon patterning homologous to those of *PIN1*; our results (Additional file 5: Figure S4) suggest that it cannot.

Finally, RPS5A::PIN1 reverted the pin-shaped, sterile inflorescences of *pin1* to WT-looking, fertile inflorescences but RPS5A::PIN6 failed to do so (Additional file 6: Figure S5), suggesting that *PIN6* is unable to provide functions in inflorescence development homologous to those of *PIN1*.

In summary, *PIN6* was unable to provide functions homologous to those of *PIN1* in control of vein network geometry, vein formation, cotyledon patterning, and inflorescence development. Thus the unequal redundancy between *PIN1* and *PIN6* in these processes is unlikely to be the result of their different expression and might instead be accounted for by their nonhomologous functions—a conclusion consistent with the opposite effects of *PIN1* and *PIN6* on intercellular auxin transport [32]. By contrast, *PIN6* was able to provide functions in vein connection homologous to those of *PIN1*, suggesting that *PIN6* expression normally limits the ability of *PIN6* to compensate for the effects of loss of *PIN1* function in vein connection.

### Homologous functions of *PIN6* and *PIN8* in *PIN1*-dependent vein-network formation

*PIN8* acts redundantly with *PIN6* in *PIN1*-dependent control of vein network geometry [33] and vein formation (Fig. 4); however, the redundancy between *PIN6* and *PIN8* in *PIN1*-dependent control of vein network formation is unequal: the geometry and cardinality index of *pin1*;*8* vein networks are no different from those of *pin1* vein networks, but those of *pin1*;*6* vein networks are; thus *PIN6* can provide all the functions of *PIN8* in *PIN1*-dependent control of vein network geometry and vein formation, but *PIN8* is unable to provide all the functions of *PIN6* in these processes. Further, *PIN8* seems to have no function in *PIN1*/*PIN6*-dependent vein connection. The unequal functions of *PIN6* and *PIN8* in vein network formation could be accounted for by the different expression of *PIN6* and *PIN8* during vein development [33] (Figs. 1 and 2), but it could also reflect nonhomologous functions of *PIN6* and *PIN8* in this process.

To test these possibilities, we expressed *PIN6* or *PIN8* by the promoter of the *MONOPTEROS* (*MP*) gene (AT1G19850) (MP::PIN6 or MP::PIN8)—highly active in developing veins [33]—in the *pin1*;*6* background, and compared defects of MP::PIN6;*pin1*;*6* and MP::PIN8;*pin1*;*6* with those of *pin1*;*6* and *pin1*.

**Fig. 7:**
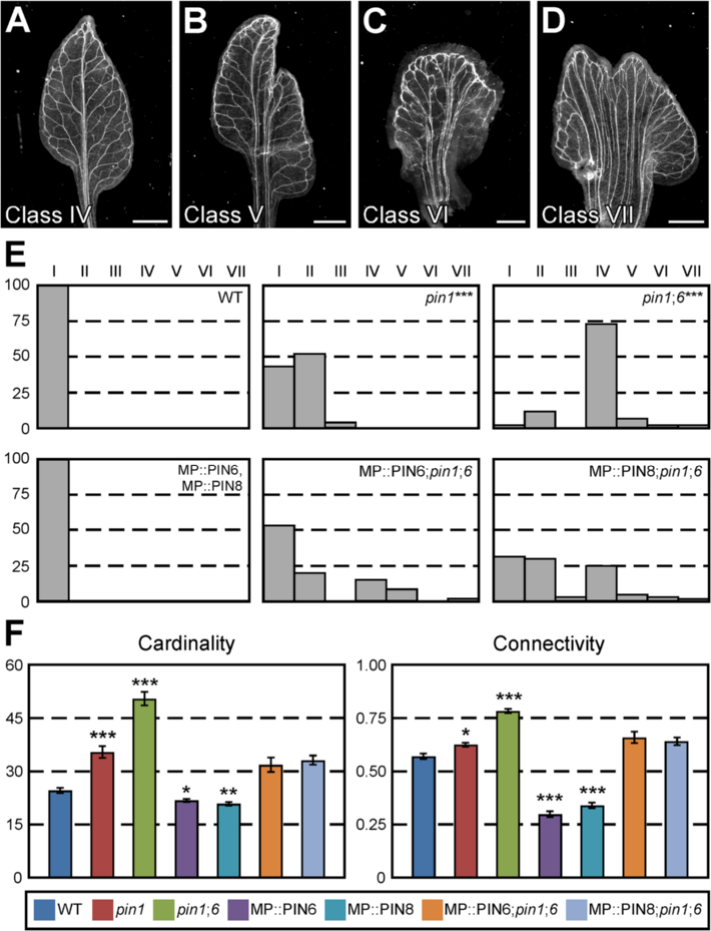
Functions of *PIN6* and *PIN8* in *PIN1*-dependent vein network formation. **a**-**d**. Dark-field illumination of mature first leaves illustrating phenotype classes: conspicuous marginal vein (**a**); fused leaves with conspicuous marginal vein (**b**); wide midvein (**c**); fused leaves with wide midvein (**d**). Phenotype classes I-III as in Fig. 6. **e**. Percentages of leaves in phenotype classes. Difference between *pin1* and WT, and between *pin1*;*6* and *pin1* was significant at *P* < 0.001 (***) by Kruskal-Wallis and Mann–Whitney test with Bonferroni correction. Sample population sizes: WT, 53; *pin1*, 46; *pin1*;*6*, 42; MP::PIN6, 54; MP::PIN8, 49; MP::PIN6;*pin1*;*6*, 45; MP::PIN8;*pin1*;*6*, 60. **f**. First leaves. Indices are expressed as mean ± SE. Difference between *pin1* and WT cardinality indices, between *pin1*;*6* and *pin1* cardinality indices, between MP::PIN6 and WT cardinality indices, between MP::PIN8 and WT cardinality indices, between *pin1* and WT connectivity indices, between *pin1*;*6* and *pin1* connectivity indices, between MP::PIN6 and WT connectivity indices, and between MP::PIN8 and WT connectivity indices was significant at *P* < 0.001 (***) by *F*-test and *t*-test with Bonferroni correction. Sample population sizes as in (**e**). Bars: (**a,b,d**) 1 mm; (**c**) 0.25 mm

We first asked whether *PIN8* could provide functions in *PIN1*-dependent control of vein network geometry homologous to those of *PIN6*. As previously reported [33], the vein network geometry of MP::PIN6 and MP::PIN8 was no different from that of WT (Fig. 7a-e). By contrast, the geometry of nearly 60 % of *pin1* vein networks was abnormal, and *pin6* shifted the spectrum of vein network geometries of *pin1* toward more severe phenotype classes [33] (Fig. 7a-e). The spectrum of vein network geometries of MP::PIN6;*pin1*;*6* was no different from that of *pin1* and that of MP::PIN8;*pin1*;*6* was no different from that of MP::PIN6;*pin1*;*6* (Fig. 7a-e), suggesting that *PIN8* can provide functions in *PIN1*-dependent control of vein network geometry homologous to those of *PIN6*.

We next asked whether *PIN8* could provide functions in *PIN1*-dependent control of vein network topology homologous to those of *PIN6*. Consistent with previous observations [33], MP::PIN6 and MP::PIN8 induced similar defects—as it frequently results from overexpression of genes with homologous functions (e.g., [78–80]): the cardinality and connectivity indices of both MP::PIN6 and MP::PIN8 vein networks were lower than those of WT vein networks (Fig. 7f), supporting that *PIN6* inhibits vein formation and connection, and suggesting that *PIN8* can inhibit vein connection in addition to vein formation. As reported above (Fig. 3b), the cardinality and connectivity indices of *pin1* vein networks were higher than those of WT vein networks and those of *pin1*;*6* vein networks were higher than those of *pin1* vein networks (Fig. 7f). The vein network topology of MP::PIN6;*pin1*;*6* was no different from that of *pin1* and that of MP::PIN8;*pin1*;*6* was no different from that of MP::PIN6;*pin1*;*6* (Fig. 7f), suggesting that *PIN8* can provide functions in *PIN1*-dependent control of vein network topology homologous to those of *PIN6*.

In addition to *PIN1*-dependent vein-network formation, *PIN8* acts redundantly with *PIN6* in *PIN1*-dependent cotyledon patterning, and as in *PIN1*-dependent vein network formation, the redundancy between *PIN6* and *PIN8* in *PIN1*-dependent cotyledon patterning is unequal [33]. We thus asked whether *PIN8* could provide functions in *PIN1*-dependent cotyledon patterning homologous to those of *PIN6*; our results (Additional file 7: Figure S6) suggest that it can.

In summary, *PIN8* was able to provide functions homologous to *PIN6* in *PIN1*-dependent vein network formation and cotyledon patterning. Thus the unequal redundancy between *PIN6* and *PIN8* is unlikely the result of nonhomologous functions and might instead be accounted for by their different expression. Just as the *ER-PIN* genes *PIN6* and *PIN8* redundantly control *PIN1*-dependent vein network formation, the redundancy between the *PM-PIN* genes *PIN1*, *PIN2*, *PIN3*, *PIN4*, and *PIN7* underlies— to varying extents—many other developmental processes (e.g., [37, 52, 81–84]). In the development of embryos and roots, *PM-PIN* genes compensate for loss of one another’s function by their ectopic expression in the domain of the gene whose function has been lost [82, 84]. For example, in *pin7* embryos PIN4 becomes expressed at earlier stages of development and in the domain in which PIN7 is normally expressed, thereby compensating for loss of *PIN7* function [84]. By contrast, in the *pin1*;*6* background *PIN8* expression remains restricted to post-formative stages of vein development [33], supporting that *PIN8* controls vein network formation by feeding back on vascular precursor cells located in more-immature parts of the leaf.

### Functions of *PIN5* in *PIN6*/*PIN8*-dependent control of vein network topology

*PIN6* has functions in control of vein network topology beyond control of *PIN5* function (Fig. 4). We asked whether *PIN5* could provide functions in control of vein network topology that are independent of control by *PIN6* or *PIN8*.

To address this question, we used plants expressing *PIN5* by the *MP* promoter (MP::PIN5) because the vein density of MP::PIN5 leaves is higher than that of WT leaves [33]. We reasoned that if *PIN5* could provide functions that are independent of control by *PIN6* or *PIN8*, at least some of the effects of MP::PIN5 on vein network topology should persist in the MP::PIN6 or MP::PIN8 backgrounds. By contrast, if all *PIN5*’s functions depended on control by *PIN6* or *PIN8*, the effects of MP::PIN6 or MP::PIN8 on vein network topology should mask those of MP::PIN5.

**Fig. 8:**
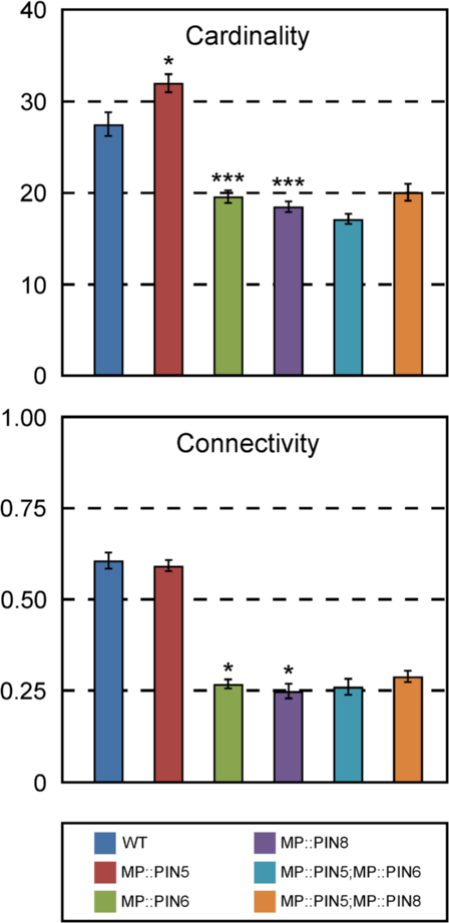
Functions of *PIN5* in *PIN6*/*PIN8*-dependent control of vein network topology. First leaves. Indices are expressed as mean ± SE. Difference between MP::PIN5 and WT cardinality indices, between MP::PIN6 and WT cardinality indices, between MP::PIN8 and WT cardinality indices, between MP::PIN6 and WT connectivity indices, and between MP::PIN8 and WT connectivity indices was significant at *P* < 0.05 (*) or *P* < 0.001 (***) by *F*-test and *t*-test with Bonferroni correction. Sample population sizes: WT, 27; MP::PIN5, 48; MP::PIN6, 32; MP::PIN8, 32; MP::PIN5;MP::PIN6, 31; MP::PIN5;MP::PIN8, 40

Consistent with previous observations [33], the cardinality index of MP::PIN5 vein networks was higher than that of WT vein networks (Fig. 8), supporting that *PIN5* promotes vein formation. As reported above (Fig. 7), the cardinality and connectivity indices of MP::PIN6 and MP::PIN8 vein networks were lower than those of WT vein networks (Fig. 8). Because the vein network topology of MP::PIN5;MP::PIN6 was no different from that of MP::PIN6 and that of MP::PIN5;MP::PIN8 was no different from that of MP::PIN8 (Fig. 8), we conclude that no function of *PIN5* escapes control by *PIN6* or *PIN8*.

## Conclusions

**Fig. 9:**
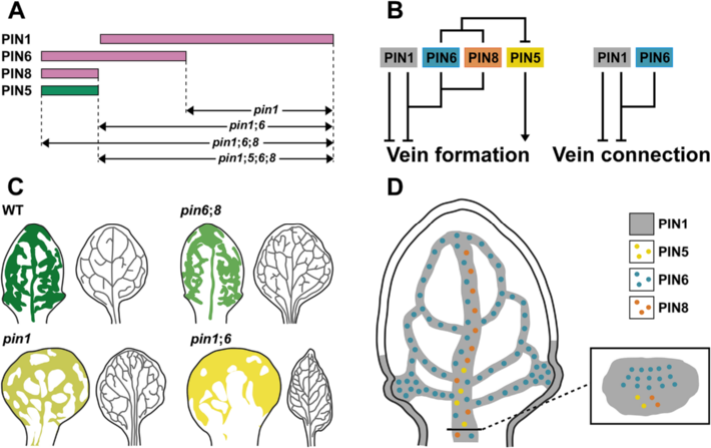
Summary and interpretations. **a**. Unique and redundant functions of *PIN1*, *PIN5*, *PIN6*, and *PIN8* in vein network formation (magenta, inhibiting functions; green, promoting functions), and derived mutant phenotypes. It is possible that *PIN8*’s functions extend to overlap with *PIN6*’s in *PIN1*-dependent inhibition of vein network formation. **b**. Genetic interaction map of *PIN1*, *PIN5*, *PIN6*, and *PIN8* in vein network topology. *Arrows* indicate positive effects; *blunt-ended lines* indicate negative effects. **c**. DR5-promoter-activity-derived auxin levels and distribution in developing leaves (*left*; lower levels are in lighter tints; for simplicity, differences within leaves are ignored), and vein networks in mature leaves (*right*). **d**. Cellular expression map of *PIN1*, *PIN5*, *PIN6*, and *PIN8* in vein development. See text for details

Vein network formation is redundantly, but nonhomologously, controlled by PIN1-mediated intercellular auxin transport and PIN6/PIN8-mediated intracellular auxin transport (Fig. 9a, b). How to account for such functional overlap?

The “auxin canalization hypothesis” proposes that a positive feedback between auxin movement through a cell and localization of auxin efflux proteins to the site where auxin leaves the cell leads to the selection of cell files from within a field of cells; such cell files would become exposed to inductive levels of auxin, would differentiate into efficient auxin-transport canals—the veins—and would drain auxin from the surrounding areas [85, 86]. Auxin drainage by developing veins would lower auxin levels in the surrounding areas below those levels which inhibit growth [87, 88], growth would resume, and new fields of cells would be generated in which the whole process could be repeated [2].

The predictions of the auxin canalization hypothesis have been rigorously tested and are supported by computer simulation of mathematical models [89–91]; nevertheless, inconsistencies seem to exist between experimental evidence and hypothesis’ predictions. For example, the hypothesis appears unable to predict the experimentally observed high levels of auxin in veins [68, 69]; however, such levels could be, at least in part, the result of PIN1-mediated auxin transport toward sites of vein formation [41] (Figs. 5 and 9d), and of PIN6/PIN8-mediated increase in auxin levels within developing vascular cells [29–33] (Fig. 5 and 9d). We suggest that because of the lower auxin levels in *pin6*;*8* [29–33] (Fig. 5), auxin would be drained more efficiently in leaves of this background, leaf growth would resume sooner, and veins would form faster—a prediction supported by the faster formation of vein-associated domains of *PIN1* expression in *pin6*;*8* [33]—thus leading to the formation of networks of more veins (Fig. 9c). Because of the reduced intercellular auxin-transport in *pin1* [92], auxin would accumulate for longer periods in leaves of this background before inducing efficient drainage canals, thereby exposing more cells to inductive levels of auxin—a prediction consistent with the broader domains of DR5rev::YFPnuc expression in *pin1* (Fig. 5)—and thus leading to the formation of networks of more veins (Fig. 9c). Because of the additionally lower levels of auxin in *pin1*;*6*—suggested by the mimicry of *pin1*;*6* defects by reduction of auxin levels in *pin1* [33] and by the lower levels of DR5rev::YFPnuc expression in *pin1*;*6* than in *pin1* (Fig. 5)—auxin would accumulate for even longer periods before inducing efficient drainage canals, thereby exposing even more cells to inductive levels of auxin—a prediction consistent with the broader domains of DR5rev::YFPnuc expression in *pin1*;*6* than in *pin1* (Fig. 5)—and thus leading to the formation of networks of even more veins (Fig. 9c).

Closed veins form during leaf development from open-vein precursors that become connected with other vein precursors at both ends [12–14, 38–40]. Accounting for the formation of closed veins has long been a challenge for the auxin canalization hypothesis (reviewed in [93–97]). Loss of function of *PIN1* and *PIN6* leads to a network of veins that are more frequently closed, and overexpression of *PIN1*, *PIN6*, or *PIN8* leads to the opposite defect; thus—consistent with the observation that vein connections form at early stages of tissue development [12–14, 38–40]—our results suggest that connection may be favored—or occur exclusively—between vein precursors that have yet to differentiate high auxin-transport capacity or high auxin-transport-mediated auxin levels.

## Methods

### Definitions, usage and notations

We define a “vein” as a stretch of vascular elements that contacts another stretch of vascular elements at least at one end (Additional file 3: Figure S2). A stretch of vascular elements that fails to satisfy this requirement is referred to as a “vein fragment” (Additional file 3: Figure S2). We refer to a vein that contacts another vein or a vein fragment only at one end as an “open” vein, and to a vein that contacts vein fragments or other veins at both ends as a “closed” vein (Additional file 3: Figure S2).

We use “vein network geometry” to indicate a set of regularities in the shape and relative position of the veins in a network—e.g., whether the midvein is I- or Y-shaped, and whether the vein network outline is scalloped or smooth—irrespective of the topology of the vein network. We use “vein network topology” to indicate a set of features of a vein network— such as the number of veins and the proportion of closed veins—that are irrespective of the geometry of the vein network.

We use “::” to indicate transcriptional fusions—i.e. fusion of, for example, promoter *A* to gene *B*—and “:” to indicate translational fusions—i.e. fusion of, for example, gene *A* to gene *B*. We use semicolons to separate mutations and transgenes in second- and higher-order combinations.

### Plants

Origin and nature of lines, genotyping strategies, and oligonucleotide sequences are in Additional file 1: Table S1, Additional file 8: Table S2, and Additional file 9: Table S3, respectively. Seeds were sterilized and germinated, and plants were grown and transformed as described in [98].

### Imaging

Developing leaves were mounted and imaged as in [33]. Marker-line-specific imaging parameters are in Additional file 10: Table S4 and Additional file 11: Table S5. Mature leaves were fixed in 3:1 ethanol:acetic acid, rehydrated in 70 % ethanol and water, cleared briefly (few seconds to few minutes) in 0.4 M sodium hydroxide, washed in water, and mounted in 1:3:8 water:glycerol:chloral hydrate (Sigma-Aldrich Co. LLC, St. Louis, MO). Mounted leaves were imaged as in [99]. Image brightness and contrast were adjusted by linear stretching of the histogram with ImageJ (National Institutes of Health, Bethesda, MD). Images were cropped with Photoshop (Adobe Systems Inc. San Jose, CA) and assembled into figures with Canvas (ACD Systems International Inc. Victoria, Canada).

### Analysis of vein network topology

We define a “touch point” (TP) as the point where a vein end contacts another vein or a vein fragment, an “end point” (EP) as the point where an open vein terminates free of contact with another vein or a vein fragment, and a “break point” (KP) as each of the two points where a vein fragment terminates free of contact with veins or other vein fragments (Additional file 3: Figure S2). Because it is impractical to determine the TP between a vein that exits a leaf and the vein system of the plant axis, or the KP in proximity of the vein system of the plant axis of a vein fragment that exits a leaf, we define an “exit point” (XP) as the point where a vein or a vein fragment exits the leaf lamina and enters the leaf petiole (Additional file 3: Figure S2), and equate an XP to a TP. The number of TPs, EPs, KPs, and XPs in dark-field images of cleared mature leaves was calculated with the Cell Counter plugin of ImageJ (National Institutes of Health).

A vein network can be understood as an undirected graph in which TPs, EPs, KPs, and XPs are vertices, and veins and vein fragments are edges. Because each vein is incident to two TPs, a TP and an XP, a TP and an EP, or an XP and an EP, the cardinality index—a measure of the size (i.e. the number of edges) of a graph—is a proxy for the number of veins and is calculated as: [(TP + XP − EP)/2] + EP, or: (TP + XP + EP)/2.

The continuity index quantifies how close a vein network is to a network with the same number of veins but in which at least one end of each vein fragment contacts a vein, and is thus calculated as the ratio of the cardinality index of the first network to the cardinality index of the second network: [(TP + XP + EP)/2]/[(TP + XP + EP + KP)/2], or: (TP + XP + EP)/(TP + XP + EP + KP).

The connectivity index quantifies how close a vein network is to a network with the same number of veins but in which both ends of each vein or vein fragment contact other veins, and is thus calculated as the ratio of the number of closed veins in the first network to the number of closed veins in the second network (i.e. the cardinality index of the second network): [(TP + XP − EP)/2]/[(TP + XP + EP + KP)/2], or: (TP + XP − EP)/(TP + XP + EP + KP).

## Additional files

Additional file 1: Table S1. Origin and nature of lines [28–31, 33–37, 53, 100]. (DOC 79 kb) Additional file 2: Figure S1. Expression of *PIN1*, *PIN6*, and *PIN8* in Arabidopsis first leaves. (A-H) Confocal laser scanning microscopy; first leaves 4 days after germination. Bottom left: reproducibility index and reporter identity. Yellow: expression of PIN1::PIN1:GFP (A), PIN1::PIN1:CFP (B), PIN6::YFPnuc (C), PIN6::CFPnuc (D), PIN6::PIN6:GFP^MGS^ (E), PIN6::PIN6:GFP^RLB^ (F), PIN8::YFPnuc (G), PIN8::PIN8:GFP^MGS^ (H), or PIN8::PIN8:GFP^ZD^ (I). Blue: autofluorescence (A,F,H,I). Dashed magenta line delineates leaf primordium outline. Boxes in (E) and (H) illustrate positions of close-ups in (F) and (I), respectively. Bars: (A-E,G,H) 100 μm; (F,I) 20 μm (TIF 4004 kb) Additional file 3: Figure S2. Analysis of vein network topology. Dark-field illumination of cleared mature first leaf (MP::PIN6) illustrating vein network elements. A “vein fragment” (magenta box) is incident to two “break points” (KPs; magenta dots)—the points where a vein fragment terminates free of contact with veins or other vein fragments. An “open vein” (blue box) is incident to a “touch point” (TP; yellow dot)—a point of contact between a vein and vein fragments or other veins—and an “end point” (EP; blue dot)—the point where an open vein terminates free of contact with another vein or a vein fragment. A “closed vein” (yellow box) is incident to two TPs. A vein or a vein fragment exits the leaf lamina and enters the leaf petiole (green box) by an “exit point” (XP; green dot). See text for details. Bar: 1 mm. (TIF 2596 kb) Additional file 4: Figure S3. Functions of *PIN1*, *PIN5*, *PIN6*, and *PIN8* in control of vein continuity. First leaves. Indices are expressed as mean ± SE. Sample population sizes as in Fig. 3B (A), Fig. 4 (B), Fig. 6D (C), Fig. 7E (D), or Fig. 8 (E). (TIF 754 kb) Additional file 5: Figure S4. Functions of *PIN1* and *PIN6* in cotyledon patterning. (A-E) Dark-field illumination of 4-day old seedlings illustrating phenotype classes: two separate cotyledons (A); fused cotyledons and separate single cotyledon (B); three separate cotyledons (C); fused cotyledons (D); single cotyledon (E). (F) Percentages of seedlings in phenotype classes. Difference between *pin1* and WT, and between RPS5A::PIN1;*pin1* and *pin1* was significant at *P* < 0.05 (*) or *P* < 0.001 (***) by Kruskal-Wallis and Mann–Whitney test with Bonferroni correction. Sample population sizes: WT, 78; RPS5A::PIN1, 68; RPS5A::PIN6, 32; *pin1*, 88; RPS5A::PIN1;*pin1*, 47; RPS5A::PIN1;*pin1*, 73. Bars: (A-C) 1 mm; (D,E) 0.5 mm. (TIF 3522 kb) Additional file 6: Figure S5. Functions of *PIN1* and *PIN6* in inflorescence development. (A-F) Four-week-old plants. Top right: genotype. Bottom left: reproducibility index. WT plants normally form fertile flowers (A), while *pin1* plants never do [92, 100] (D). Bars: 5 mm. (TIF 1180 kb) Additional file 7: Figure S6. Functions of *PIN6* and *PIN8* in *PIN1*-dependent cotyledon patterning. (A,B) Dark-field illumination of 4-day old seedlings illustrating phenotype classes: partially fused cup-shaped cotyledons, side view; inset: top view (A); completely fused cup-shaped cotyledon, side view; inset: top view (B). Phenotype classes a-e as in Additional file 5: Figure S4. (C) Percentages of seedlings in phenotype classes. Difference between *pin1* and WT, between *pin1*;*6* and *pin1*, and between MP::PIN6;*pin1*;*6* and *pin1*;*6* was significant at *P* < 0.01 (**) or *P* < 0.001 (***) by Kruskal-Wallis and Mann–Whitney test with Bonferroni correction. Sample population sizes: WT, 53; *pin1*, 52; *pin1*;*6*, 55; MP::PIN6, 54; MP::PIN8, 49; MP::PIN6;*pin1*;*6*, 47; MP::PIN8;*pin1*;*6*, 62. Bars: (A) 0.5 mm; (B) 0.25 mm. (TIF 1181 kb) Additional file 8: Table S2. Genotyping strategies. (DOC 43 kb) Additional file 9: Table S3. Oligonucleotide sequences. (DOC 63 kb) Additional file 10: Table S4. Imaging parameters: single-marker lines. (DOC 50 kb) Additional file 11: Table S5. Imaging parameters: double-marker lines. (DOC 85 kb)

## Abbreviations

CFPnuc: Nuclear cyan fluorescent protein
DAG: Days after germination
DR5rev: DIRECT REPEAT5 reverse
ER: Endoplasmic reticulum
GFP: Green fluorescent protein
MP: MONOPTEROS
PIN: PIN-FORMED
PM: Plasma membrane
RPS5A: RIBOSOMAL PROTEIN S5A
YFPnuc: Nuclear yellow fluorescent protein
