# Supplementary material for: Control of vein network topology by auxin transport
Source: BMC Biol. 2015 Nov 11;13:94. doi: 10.1186/s12915-015-0208-3 (PMC4641347; doi:10.1186/s12915-015-0208-3)
Supplement: Additional file 1: Table S1. — Origin and nature of lines [28–31, 33–37, 53, 100]. (DOC 79 kb) [file 12915_2015_208_MOESM1_ESM.doc]

**Table S1. Origin and nature of lines.**

| **Line** | **Origin/Nature** |
| --- | --- |
| PIN1::PIN1:GFP | [37] |
| PIN1:PIN1:CFP | [53] (ABRC); introgressed into Col-0 |
| PIN6::YFPnuc | [33] |
| PIN6::CFPnuc | Transcriptional fusion of *PIN6* (AT1G77110; -3784 to -1; primers: ‘PIN6 prom SalI F’ and ‘PIN6 prom BamHI R’) to ECFP:3xNLS (Clontech Laboratories Inc.) |
| PIN6::PIN6:GFP^MGS^ | [33] |
| PIN6::PIN6:GFP^RLB^ | [31] |
| PIN8::YFPnuc | [33] |
| PIN8::PIN8:GFP^MGS^ | [33] |
| PIN8::PIN8:GFP^ZD^ | [30] |
| PIN5::YFPnuc | Transcriptional fusion of *PIN5* (AT5G16530; -3279 to -3; primers: ‘PIN5 SpeI KpnI transc forw’ and ‘PIN5 AgeI transc rev’) to EYFP:3xNLS (Clontech Laboratories Inc.) |
| PIN5::PIN5:GFP^MGS^ | Translational fusion of *PIN5* (AT5G16530; -4311 to +3606; primers: ‘PIN5 extra prom XhoI forw’ and ‘PIN5 extra prom SalI rev’, ‘PIN5 prom XhoI forw’ and PIN5 4991 BamHI rev’, ‘PIN5 4992 XbaI forw’ and ‘PIN5 UTR SacI rev’, ‘PIN5 extra UTR SmaI forw’ and ‘PIN5 extra UTR SacI rev’) to EGFP (Clontech Laboratories Inc.; insertion at +1712 of *PIN5*; primers: ‘EGFP BamHI forw’ and ‘EGFP XbaI rev’); reverts the cotyledon phenotype of *pin1*;*5*;*6*;*8* to that of *pin1*;*6*;*8* |
| PIN5:PIN5:GFP^AG^ | [34] |
| DR5rev::YFPnuc | [33, 36] |
| *pin1-1* | [33, 35, 100]; WT at the *TTG1* (AT5G24520) locus; contains a G-to-A transition at position +431, resulting in a stop codon after amino acid 143 |
| *pin5-4* | [28] |
| *pin6* | [33] |
| *pin8-1* | [29] |
| RPS5A::PIN1 | Kind gift of P. Dhonukshe |
| RPS5A::PIN6 | [33] |
| MP::PIN6 | [33] |
| MP::PIN8 | [33] |
| MP::PIN5 | [33] |
