# Supplementary figures and images for: Control of vein network topology by auxin transport
Source: BMC Biol. 2015 Nov 11;13:94. doi: 10.1186/s12915-015-0208-3 (PMC4641347; doi:10.1186/s12915-015-0208-3)

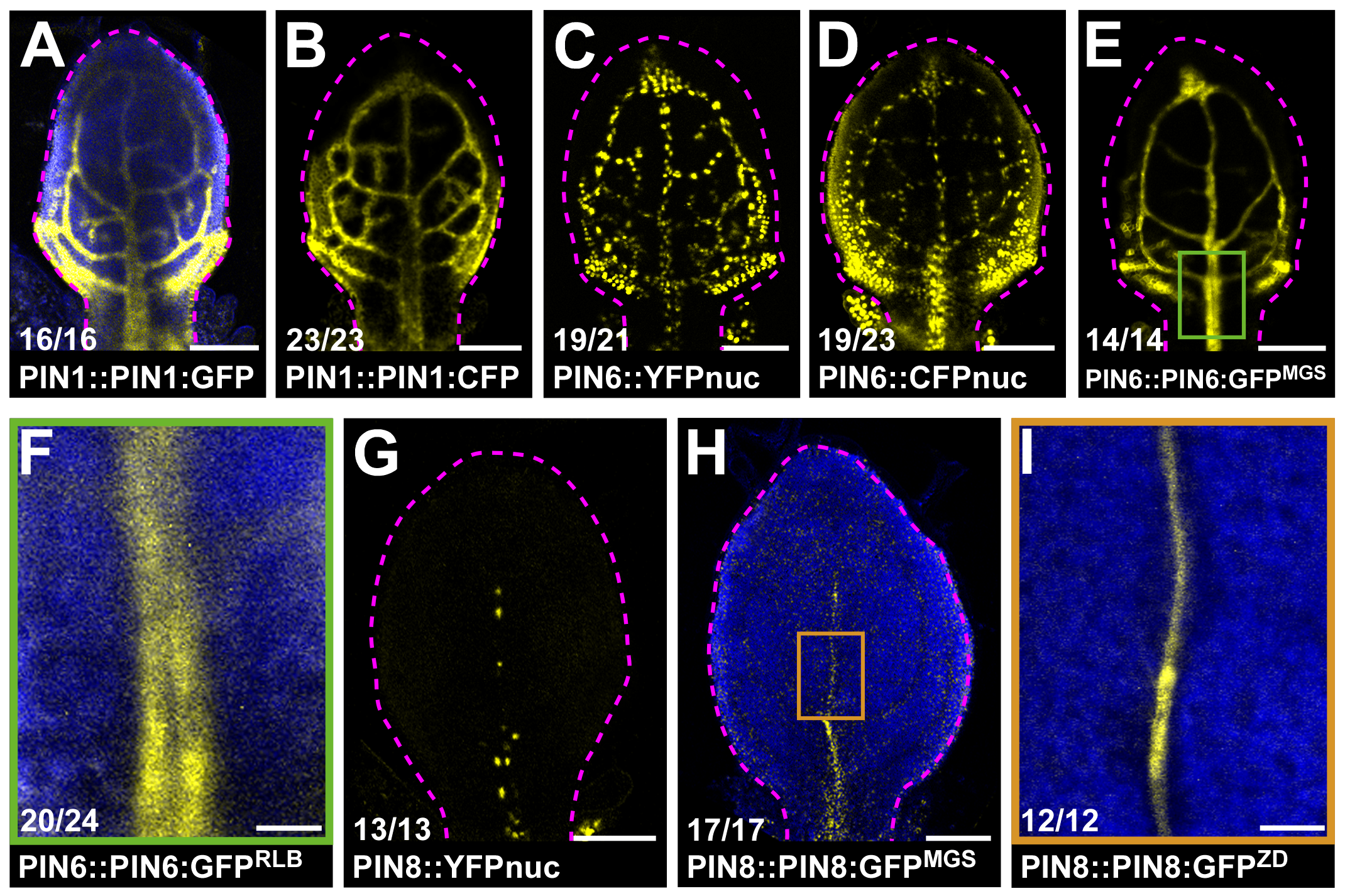

Supplement: Additional file 2: Figure S1. — Expression of PIN1, PIN6, and PIN8 in Arabidopsis first leaves. (A-H) Confocal laser scanning microscopy; first leaves 4 days after germination. Bottom left: reproducibility index and reporter identity. Yellow: expression of PIN1::PIN1:GFP (A), PIN1::PIN1:CFP (B), PIN6::YFPnuc (C), PIN6::CFPnuc (D), PIN6::PIN6:GFPMGS (E), PIN6::PIN6:GFPRLB (F), PIN8::YFPnuc (G), PIN8::PIN8:GFPMGS (H), or PIN8::PIN8:GFPZD (I). Blue: autofluorescence (A,F,H,I). Dashed magenta line delineates leaf primordium outline. Boxes in (E) and (H) illustrate positions of close-ups in (F) and (I), respectively. Bars: (A-E,G,H) 100 μm; (F,I) 20 μm (TIF 4004 kb) [file 12915_2015_208_MOESM2_ESM.tif]

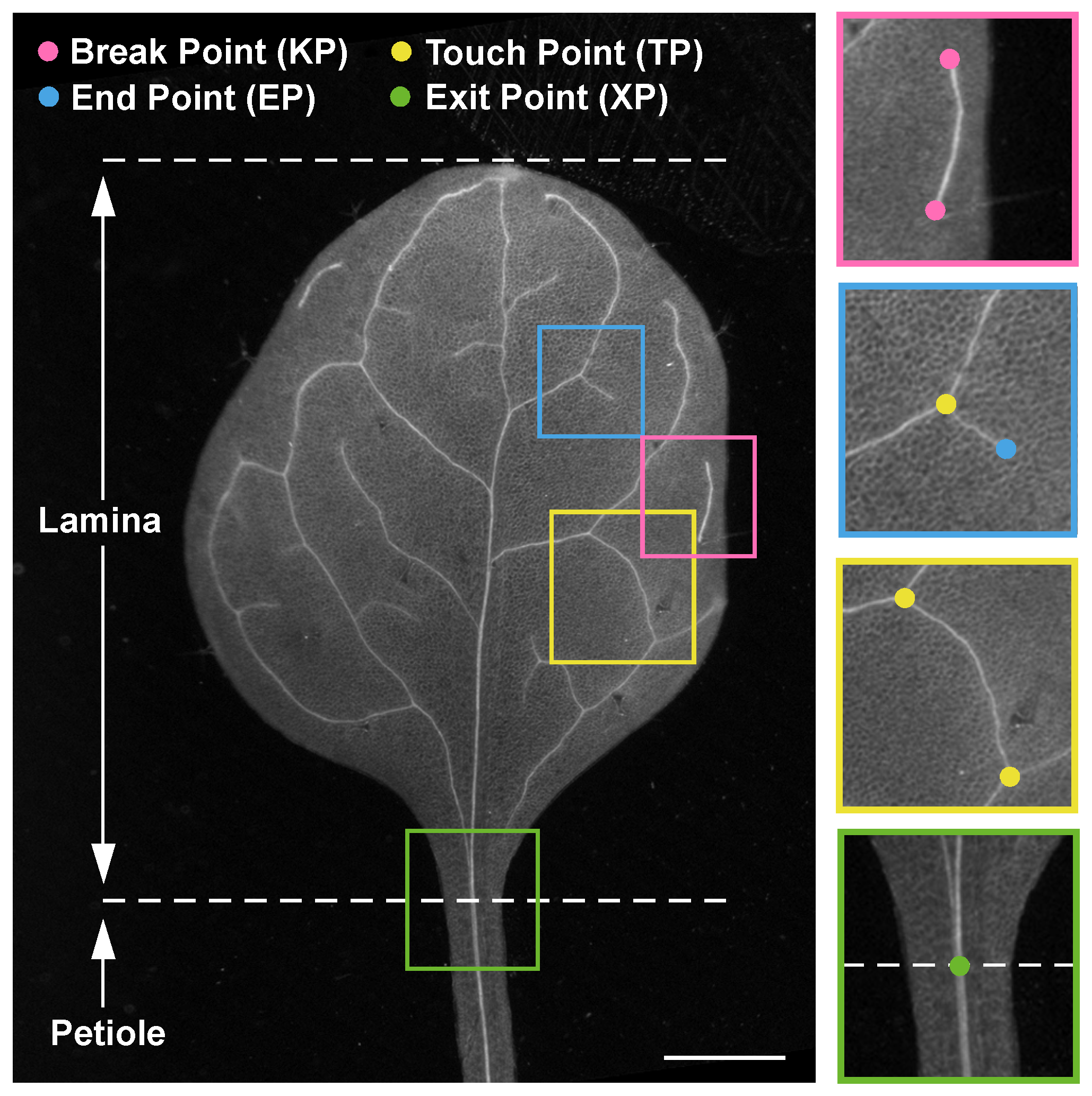

Supplement: Additional file 3: Figure S2. — Analysis of vein network topology. Dark-field illumination of cleared mature first leaf (MP::PIN6) illustrating vein network elements. A “vein fragment” (magenta box) is incident to two “break points” (KPs; magenta dots)—the points where a vein fragment terminates free of contact with veins or other vein fragments. An “open vein” (blue box) is incident to a “touch point” (TP; yellow dot)—a point of contact between a vein and vein fragments or other veins—and an “end point” (EP; blue dot)—the point where an open vein terminates free of contact with another vein or a vein fragment. A “closed vein” (yellow box) is incident to two TPs. A vein or a vein fragment exits the leaf lamina and enters the leaf petiole (green box) by an “exit point” (XP; green dot). See text for details. Bar: 1 mm. (TIF 2596 kb) [file 12915_2015_208_MOESM3_ESM.tif]

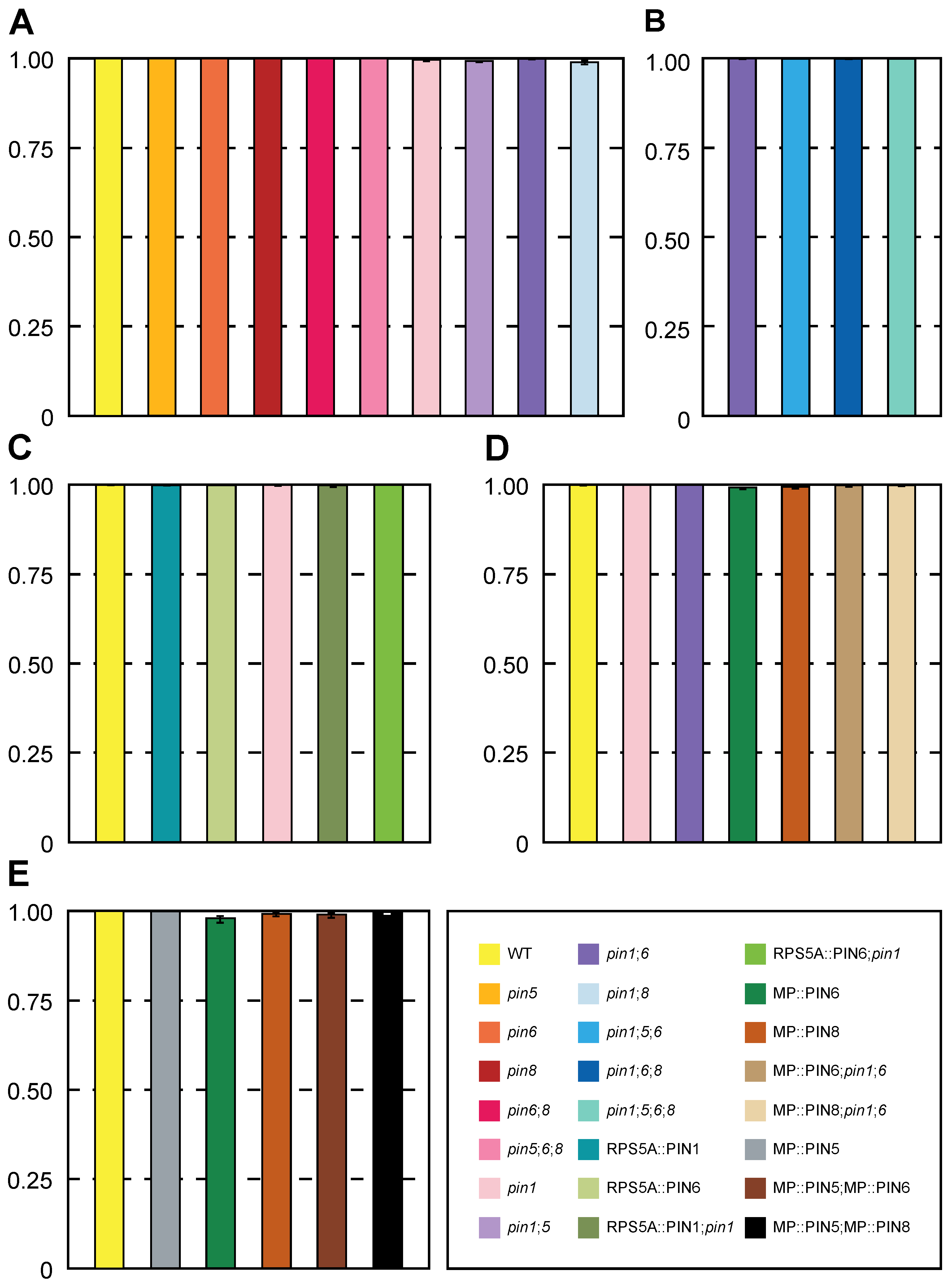

Supplement: Additional file 4: Figure S3. — Functions of PIN1, PIN5, PIN6, and PIN8 in control of vein continuity. First leaves. Indices are expressed as mean ± SE. Sample population sizes as in Fig. 3B (A), Fig. 4 (B), Fig. 6D (C), Fig. 7E (D), or Fig. 8 (E). (TIF 754 kb) [file 12915_2015_208_MOESM4_ESM.tif]

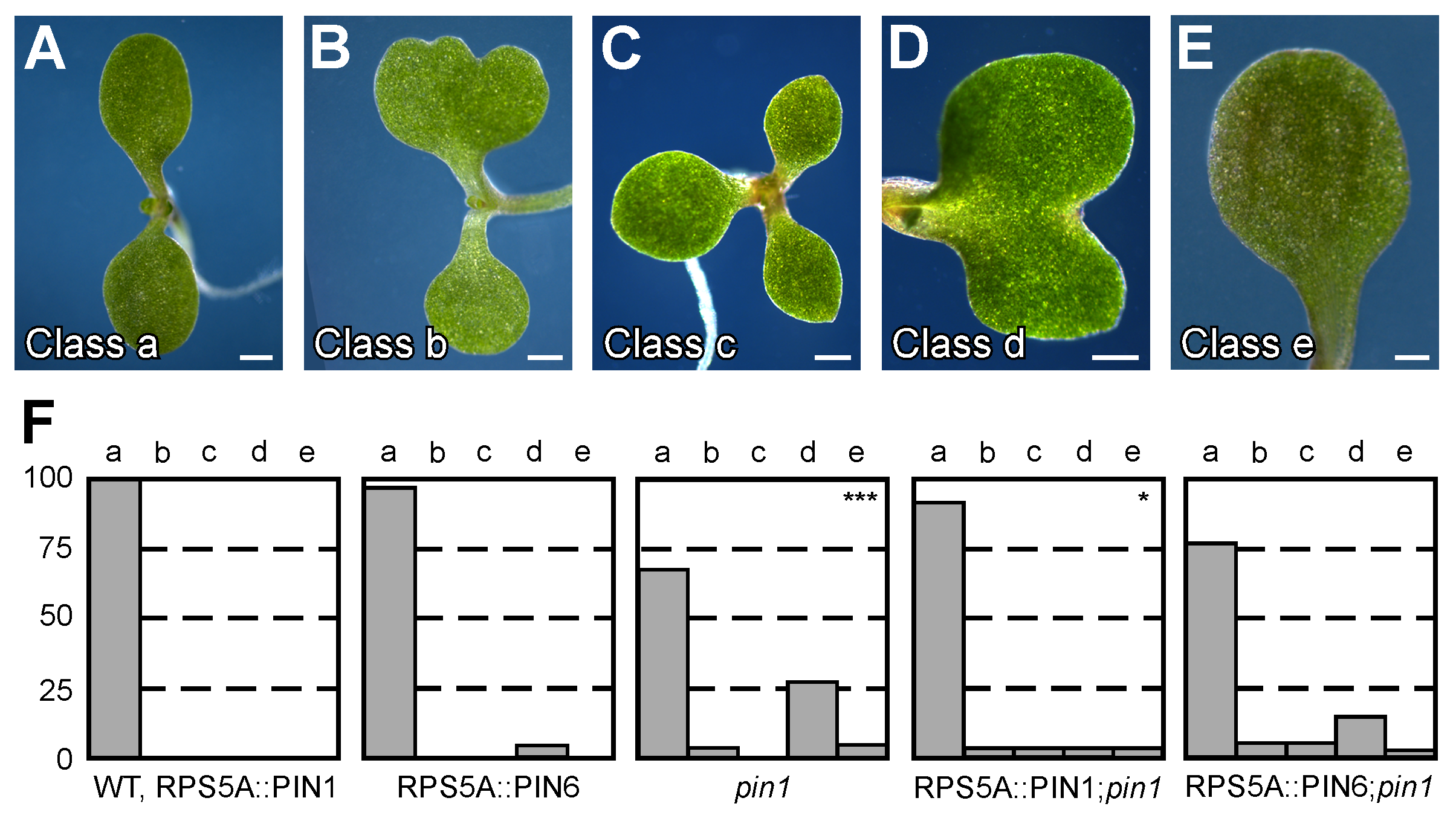

Supplement: Additional file 5: Figure S4. — Functions of PIN1 and PIN6 in cotyledon patterning. (A-E) Dark-field illumination of 4-day old seedlings illustrating phenotype classes: two separate cotyledons (A); fused cotyledons and separate single cotyledon (B); three separate cotyledons (C); fused cotyledons (D); single cotyledon (E). (F) Percentages of seedlings in phenotype classes. Difference between pin1 and WT, and between RPS5A::PIN1;pin1 and pin1 was significant at P < 0.05 (*) or P < 0.001 (***) by Kruskal-Wallis and Mann–Whitney test with Bonferroni correction. Sample population sizes: WT, 78; RPS5A::PIN1, 68; RPS5A::PIN6, 32; pin1, 88; RPS5A::PIN1;pin1, 47; RPS5A::PIN1;pin1, 73. Bars: (A-C) 1 mm; (D,E) 0.5 mm. (TIF 3522 kb) [file 12915_2015_208_MOESM5_ESM.tif]

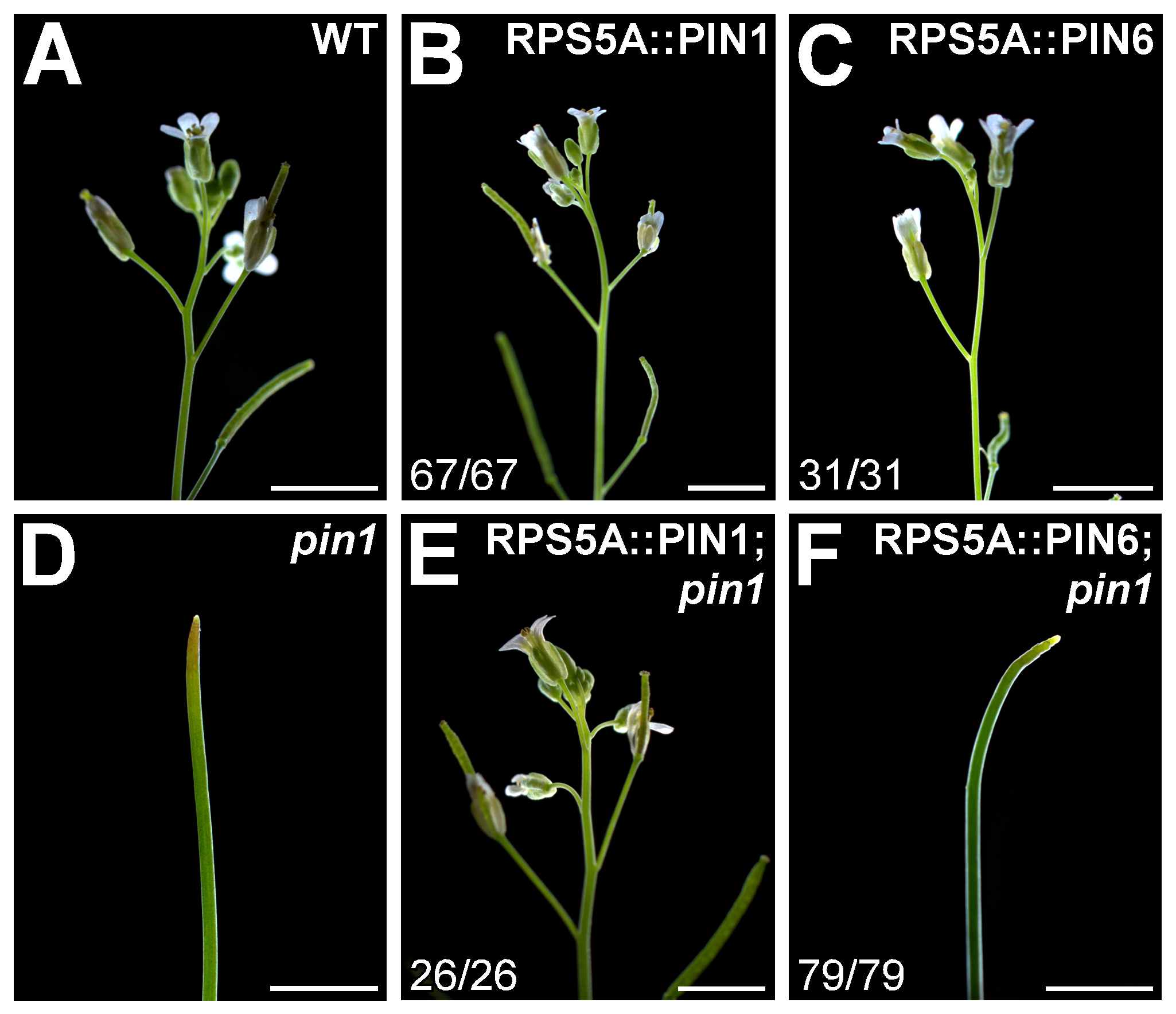

Supplement: Additional file 6: Figure S5. — Functions of PIN1 and PIN6 in inflorescence development. (A-F) Four-week-old plants. Top right: genotype. Bottom left: reproducibility index. WT plants normally form fertile flowers (A), while pin1 plants never do [92, 100] (D). Bars: 5 mm. (TIF 1180 kb) [file 12915_2015_208_MOESM6_ESM.tif]

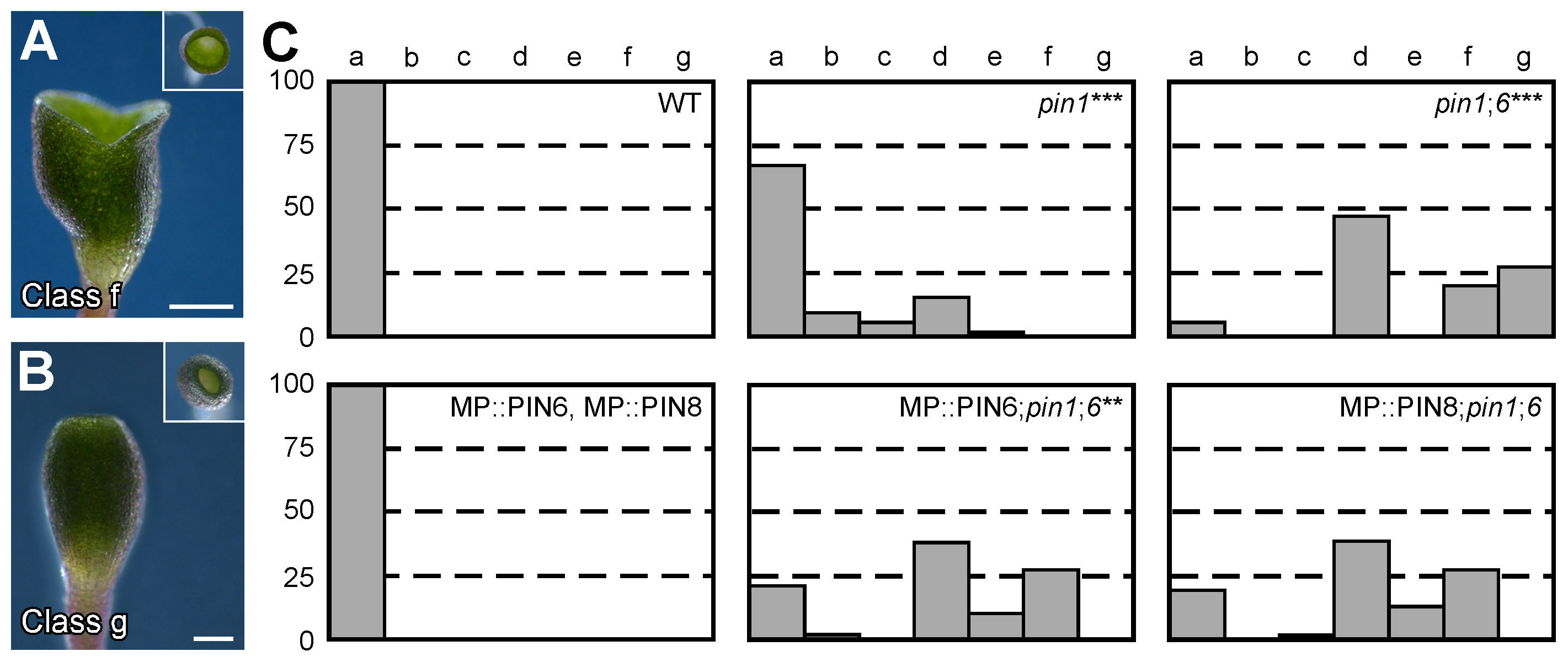

Supplement: Additional file 7: Figure S6. — Functions of PIN6 and PIN8 in PIN1-dependent cotyledon patterning. (A,B) Dark-field illumination of 4-day old seedlings illustrating phenotype classes: partially fused cup-shaped cotyledons, side view; inset: top view (A); completely fused cup-shaped cotyledon, side view; inset: top view (B). Phenotype classes a-e as in Additional file 5: Figure S4. (C) Percentages of seedlings in phenotype classes. Difference between pin1 and WT, between pin1;6 and pin1, and between MP::PIN6;pin1;6 and pin1;6 was significant at P < 0.01 (**) or P < 0.001 (***) by Kruskal-Wallis and Mann–Whitney test with Bonferroni correction. Sample population sizes: WT, 53; pin1, 52; pin1;6, 55; MP::PIN6, 54; MP::PIN8, 49; MP::PIN6;pin1;6, 47; MP::PIN8;pin1;6, 62. Bars: (A) 0.5 mm; (B) 0.25 mm. (TIF 1181 kb) [file 12915_2015_208_MOESM7_ESM.tif]
