# Supplementary material for: Control of vein network topology by auxin transport
Source: BMC Biol. 2015 Nov 11;13:94. doi: 10.1186/s12915-015-0208-3 (PMC4641347; doi:10.1186/s12915-015-0208-3)
Supplement: Additional file 8: Table S2. — Genotyping strategies. (DOC 43 kb) [file 12915_2015_208_MOESM8_ESM.doc]

**Table S2. Genotyping strategies.**

| **Line** | **Strategy** |
| --- | --- |
| *pin1-1* | In RPS5A::PIN1 background: ‘0.28 PIN1p SalI’ and ‘pin1-1 R’, and *Tat*I. In all other backgrounds: ‘pin1-1 F’ and ‘pin1-1 R’, and *Tat*I; or ‘Pin1-1 WT KpnI Fwd’ and ‘Pin1-1 WT KpnI Rev’, and *Kpn*I |
| *pin5-4* | *PIN5*: ‘SALK_042994 LP’ and ‘SALK_042994 RP’; *pin5*: ‘SALK_042994 RP’ and ‘LBb1.3’ |
| *pin6* | *PIN6* (in MP::PIN6 background): ‘PIN6 prom seq forw’ and ‘PIN6 spm R’; *PIN6* (in all other backgrounds): ‘PIN6 spm F’ and ‘PIN6 spm R’; *pin6*: ‘PIN6 spm F’ and ‘Spm32’ |
| *pin8-1* | *PIN8*: ‘SALK_107965 LP’ and ‘SALK_107965 RP’; *pin8*: ‘SALK_107965 RP’ and ‘LBb1.3’ |
| RPS5A::PIN6 | ‘PIN6 ox SmaI forw’ and ‘PIN6 ox Ecl136II rev’ |
| MP::PIN6 | ‘PIN6 ox SmaI forw’ and ‘PIN6 ox Ecl136II rev’ |
| MP::PIN8 | ‘SALK_107965 RP’ and ‘WiscDsLox489-492C10 RP’ |
| MP::PIN5 | ‘PIN5 ox SmaI forw’ and ‘PIN5 ox BamHI rev 2’ |
