# Supplementary material for: Control of vein network topology by auxin transport
Source: BMC Biol. 2015 Nov 11;13:94. doi: 10.1186/s12915-015-0208-3 (PMC4641347; doi:10.1186/s12915-015-0208-3)
Supplement: Additional file 9: Table S3. — Oligonucleotide sequences. (DOC 63 kb) [file 12915_2015_208_MOESM9_ESM.doc]

**Table S3. Oligonucleotide sequences.**

| **Name** | **Sequence (5' to 3')** |
| --- | --- |
| PIN5 SpeI KpnI transc forw | ATAACTAGTGGTACCGAGAGAGAGAGAGAGAGAC |
| PIN5 AgeI transc rev | CTCACCGGTTTTTATCAGAAAAATAGAAATGTTGCAG |
| PIN5 extra prom XhoI forw | ATACTCGAGAGCAGGCGAATCAGGAAGATCAAC |
| PIN5 extra prom SalI rev | ATTGTCGACTCTCTCTCTCTCTCTCTCTCTCTCTCTC |
| PIN5 prom XhoI forw | ATTCTCGAGACGGAGAAAGGAGAAGAAC |
| PIN5 4991 BamHI rev | ATAGGATCCTCCATATTCCTTAGCAAAAATGAAC |
| PIN5 4992 XbaI forw | ATGTCTAGATTACATGCAGATGTTCTAAG |
| PIN5 UTR SacI rev | ATAGAGCTCATTCGGTTGAGGAGAAAG |
| PIN5 extra UTR SmaI forw | ATTCCCGGGCAATATGAAGATAAAAGGG |
| PIN5 extra UTR SacI rev | ATAGAGCTCAGCCACGAACAGAAACCATCAAG |
| EGFP BamHI forw | TATGGATCCGTGAGCAAGGGCGAGGAG |
| EGFP XbaI rev | TATTCTAGACTTGTACAGCTCGTCCATGC |
| PIN6 prom SalI F | GCGGTCGACTGATGATTGTTTAAGATAAG |
| PIN6 prom BamHI R | TCTGGATCCTCTTTGCCTCTTCTTCTTC |
| 0.28 PIN1p SalI | GCGGTCGACTACACGTAAACTACTTTTG |
| pin1-1 R | TTCCGACCACCACCAGAAGCC |
| pin1-1 F | ATGATTACGGCGGCGGACTTCTA |
| Pin1-1 WT KpnI Fwd | TTCTCTCTCTCGACACTCCCC |
| Pin1-1 WT KpnI Rev | CAAAGAGAAAGAGCATGAGTGGGTAC |
| SALK_042994 LP | TGTGGTTGTGGGAGAGAAGTC |
| SALK_042994 RP | AAATTTGGACTTACGCTGTGC |
| LBb1.3 | ATTTTGCCGATTTCGGAAC |
| PIN6 prom seq forw | GGTAATCTCGTCAACAAGTCTC |
| PIN6 spm R | GGAGTTCAAAGAGGAATAGTAGCAGAG |
| PIN6 spm F | CATAACGAAGCTAACTAAGGGGTAATCTC |
| Spm32 | TACGAATAAGAGCGTCCATTTTAGAGTG |
| SALK_107965 LP | TGAAAGACATTTTGATGGCATC |
| SALK_107965 RP | CCAAATCAAGCTTTGCAAGAC |
| PIN6 ox SmaI forw | ATACCCGGGATGATAACGGGAAACGAATTCTAC |
| PIN6 ox Ecl136II rev | ATTGAGCTCTCATAGGCCCAAGAGGACG |
| WiscDsLox489-492C10 RP | TTGGAAAGGAAAAGAACACCC |
| PIN5 ox SmaI forw | ATACCCGGGATGATAAATTGTGGAGA |
| PIN5 ox BamHI rev 2 | ATTGGATCCTCAATGAATAAACTCCAGAGC |
