# Supplementary material for: Control of vein network topology by auxin transport
Source: BMC Biol. 2015 Nov 11;13:94. doi: 10.1186/s12915-015-0208-3 (PMC4641347; doi:10.1186/s12915-015-0208-3)
Supplement: Additional file 10: Table S4. — Imaging parameters: single-marker lines. (DOC 50 kb) [file 12915_2015_208_MOESM10_ESM.doc]

**Table S4. Imaging parameters: single-marker lines.**

| **Line** | **Laser** | **Wavelength (nm)** | **Main dichroic beam splitter** | **First secondary dichroic beam splitter** | **Second secondary dichroic beam splitter** | **Emission filter (detector)** |
| --- | --- | --- | --- | --- | --- | --- |
| PIN1::PIN1:CFP | Ar | 458 | HFT 458/543 | NFT 595 | NFT 545 | BP 475-525 |
| PIN6::YFPnuc | Ar | 514 | HFT 405/514/594 | NFT 595 | NFT 515 | BP 520-555 IR (PMT3) |
| PIN6::CFPnuc | Ar | 458 | HFT 458/543 | NFT 595 | NFT 545 | BP 475-525 |
| PIN8::YFPnuc | Ar | 514 | HFT 405/514/594 | NFT 595 | NFT 515 | BP 520-555 IR (PMT3) |
| PIN1::PIN1:GFP | Ar | 488 | HFT 405/488/594 | NFT 545 | NFT 490 | BP 505-530 (PMT3) |
| PIN5::YFPnuc | Ar | 514 | HFT 405/514/594 | NFT 595 | NFT 515 | BP 520-555 IR (PMT3) |
| DR5rev::YFPnuc | Ar | 514 | HFT 405/514/594 | NFT 595 | NFT 515 | BP 520-555 IR (PMT3) |
