# Supplementary material for: Control of vein network topology by auxin transport
Source: BMC Biol. 2015 Nov 11;13:94. doi: 10.1186/s12915-015-0208-3 (PMC4641347; doi:10.1186/s12915-015-0208-3)
Supplement: Additional file 11: Table S5. — Imaging parameters: double-marker lines. (DOC 85 kb) [file 12915_2015_208_MOESM11_ESM.doc]

**Table S5. Imaging parameters: double-marker lines.**

| **Double-marker lines** | **Single-marker lines** | **Laser** | **Wavelength (nm)** | **Main dichroic beam splitter** | **First secondary dichroic beam splitter** | **Second secondary dichroic beam splitter** | **Emission filter (detector)** |
| --- | --- | --- | --- | --- | --- | --- | --- |
| PIN1::PIN1:GFP;  autofluorescence | PIN1::PIN1:GFP | Ar | 488 | HFT 405/488/594 |  |  | 507-593 (META) |
|  | Autofluorescence | Ar | 488 | HFT 405/488/594 |  |  | 507-593 (META) |
| PIN6::PIN6:GFP^MGS^;  autofluorescence | PIN6::PIN6:GFP^MGS^ | Ar | 488 | HFT 405/488/594 |  |  | 507-593 (META) |
|  | Autofluorescence | Ar | 488 | HFT 405/488/594 |  |  | 507-593 (META) |
| PIN6::PIN6:GFP^RLB^;  autofluorescence | PIN6::PIN6:GFP^RLB^ | Ar | 488 | HFT 405/488/594 | Mirror | NFT 545 | BP 505-530 (PMT2) |
|  | Autofluorescence | Ar | 488 | HFT 405/488/594 | Mirror | NFT 545 | BP 600-650 (PMT3) |
| PIN8::PIN8:GFP^MGS^;  autofluorescence | PIN5::PIN5:GFP | Ar | 488 | HFT 405/488/594 |  |  | 507-593 (META) |
|  | Autofluorescence | Ar | 488 | HFT 405/488/594 |  |  | 507-593 (META) |
| PIN8::PIN8:GFP^ZD^;  autofluorescence | PIN8::PIN8:GFP^ZD^ | Ar | 488 | HFT 405/488/594 | Mirror | NFT 545 | BP 505-530 (PMT2) |
|  | Autofluorescence | Ar | 488 | HFT 405/488/594 | Mirror | NFT 545 | BP 600-650 (PMT3) |
| PIN5::PIN5:GFP;  autofluorescence | PIN5::PIN5:GFP | Ar | 488 | HFT 405/488/594 |  |  | 507-593 (META) |
|  | Autofluorescence | Ar | 488 | HFT 405/488/594 |  |  | 507-593 (META) |
| PIN1::PIN1:GFP;  PIN5::YFPnuc | PIN1::PIN1:GFP | Ar | 488 | HFT 405/488/594 |  |  | 507-593 (META) |
|  | PIN5::YFPnuc | Ar | 488 | HFT 405/488/594 |  |  | 507-593 (META) |
| PIN1::PIN1:GFP;  PIN6::YFPnuc | PIN1::PIN1:GFP | Ar | 488 | HFT 405/488/594 |  |  | 507-593 (META) |
|  | PIN6::YFPnuc | Ar | 488 | HFT 405/488/594 |  |  | 507-593 (META) |
| PIN1::PIN1:GFP;  PIN8::YFPnuc | PIN1::PIN1:GFP | Ar | 488 | HFT 405/488/594 |  |  | 507-593 (META) |
|  | PIN8::YFPnuc | Ar | 488 | HFT 405/488/594 |  |  | 507-593 (META) |
| PIN6::CFPnuc;  PIN5::YFPnuc | PIN6::CFPnuc | Ar | 458 | HFT 458/514 | NFT 595 | NFT 545 | BP 475-525 (PMT2) |
|  | PIN5::YFPnuc | Ar | 514 | HFT 458/514 | NFT 595 | NFT 515 | BP 520-355 IR (PMT3) |
| PIN8::PIN8:GFP; PIN5::YFPnuc | PIN8::PIN8:GFP | Ar | 488 | HFT 405/488/594 |  |  | 507-593 (META) |
|  | PIN5::YFPnuc | Ar | 488 | HFT 405/488/594 |  |  | 507-593 (META) |
| PIN6::CFPnuc;  PIN8::YFPnuc | PIN6::CFPnuc | Ar | 458 | HFT 458/514 | NFT 595 | NFT 545 | BP 475-525 (PMT2) |
|  | PIN8::YFPnuc | Ar | 514 | HFT 458/514 | NFT 595 | NFT 515 | BP 520-355 (PMT3) |
